# MiR-192-Mediated Positive Feedback Loop Controls the Robustness of Stress-Induced p53 Oscillations in Breast Cancer Cells

**DOI:** 10.1371/journal.pcbi.1004653

**Published:** 2015-12-07

**Authors:** Richard Moore, Hsu Kiang Ooi, Taek Kang, Leonidas Bleris, Lan Ma

**Affiliations:** 1 Bioengineering Department, University of Texas at Dallas, Richardson, Texas, United States of America; 2 Center for Systems Biology, University of Texas at Dallas, Richardson, Texas, United States of America; 3 Electrical Engineering Department, University of Texas at Dallas, Richardson, Texas, United States of America; ETH Zurich, SWITZERLAND

## Abstract

The p53 tumor suppressor protein plays a critical role in cellular stress and cancer prevention. A number of post-transcriptional regulators, termed microRNAs, are closely connected with the p53-mediated cellular networks. While the molecular interactions among p53 and microRNAs have emerged, a systems-level understanding of the regulatory mechanism and the role of microRNAs-forming feedback loops with the p53 core remains elusive. Here we have identified from literature that there exist three classes of microRNA-mediated feedback loops revolving around p53, all with the nature of positive feedback coincidentally. To explore the relationship between the cellular performance of p53 with the microRNA feedback pathways, we developed a mathematical model of the core p53-MDM2 module coupled with three microRNA-mediated positive feedback loops involving miR-192, miR-34a, and miR-29a. Simulations and bifurcation analysis in relationship to extrinsic noise reproduce the oscillatory behavior of p53 under DNA damage in single cells, and notably show that specific microRNA abrogation can disrupt the wild-type cellular phenotype when the ubiquitous cell-to-cell variability is taken into account. To assess these *in silico* results we conducted microRNA-perturbation experiments in MCF7 breast cancer cells. Time-lapse microscopy of cell-population behavior in response to DNA double-strand breaks, together with image classification of single-cell phenotypes across a population, confirmed that the cellular p53 oscillations are compromised after miR-192 perturbations, matching well with the model predictions. Our study via modeling in combination with quantitative experiments provides new evidence on the role of microRNA-mediated positive feedback loops in conferring robustness to the system performance of stress-induced response of p53.

## Author Summary

DNA damage triggered activities of the tumor suppressor protein p53 could be significantly dynamical. The functional role of p53 oscillations in cellular decision making during cancer development has been appreciated. A set of recent studies have revealed extensive crosstalk between the p53 network and microRNAs, but the specifics of the participation of microRNAs in the regulation of the p53 signaling pathway remains largely elusive. Here we investigated microRNAs that form feedback regulation with p53. We enumerated the molecular interactions among these microRNAs and the p53 core and developed a mathematical model to reproduce the DNA damage induced p53 oscillations in single cells. We performed computer simulations and system analysis in combination with experimental assessment to probe the behavior of p53 under microRNA-inhibited conditions. We show that the robust cellular performance of the stress response of p53 in a breast cancer cell line is controlled by miR-192, which forms positive feedback loops with p53.

## Introduction

Cells depend on complex intracellular signaling to process and react to external stimuli. One prominent type of dynamic response is the periodic accumulation of key transcription factors in the nucleus, where they elicit temporally controlled gene activation [1–4]. The tumor suppressor protein p53, a pivotal player involved in cancer initiation and prevention [5], undergoes oscillations in response to extracellular stress signals. Experiments show that transient DNA lesion of double-strand breaks, induced by acute application of γ-irradiation, trigger oscillatory response of the p53 protein and its negative regulator MDM2 [6–8]. At a single-cell level, the oscillation of p53 is undamped and the mean period of the pulses are constant and independent on the damage level [7]. While the cellular function of the oscillatory dynamics of these transcription factors is unclear, investigations have started to probe the significance of the p53 oscillations in inducing downstream effect such as apoptosis. For instance, recent results demonstrate that the dynamical pattern and not the absolute level of p53 protein controls the life-or-death fate decision in response to DNA damage at cellular level, highlighting the role of p53 oscillations in cellular decision making in cancer [9, 10].

Negative feedback has the potential to generate limit-cycle oscillations and is viewed as a necessary structure for biochemical oscillators [11, 12]. Indeed there exists a consensus in the literature that the p53-MDM2 negative autoregulatory loop is essential for the stress-induced p53 oscillations [3, 13]. A number of mathematical models, that typically assume an explicit time delay in the core p53-MDM2 autoregulatory loop, can reproduce the undamped p53 oscillations [14–16]. More generally, coupled negative and positive feedback loops can give rise to oscillatory phenotypes [11, 17]. The architecture of positive feedback loops, on top of a negative feedback loop, can endow performance properties such as the tunability of frequency, entrainability to cycles, and robustness under molecular noise [17–19]. Indeed, mathematical models can predict sustained oscillations under auxiliary positive feedback loops on p53 [20], but the general role of positive feedback loops in p53 oscillations remains largely elusive.

MicroRNAs are small noncoding RNAs, approximately 22 nucleotides in length that serve as post-transcriptional regulators, and have been shown to regulate the activity of nearly 30% of all protein-coding genes. Intriguingly, a set of recent studies revealed extensive crosstalk between the p53 network and microRNAs [21, 22]. We have identified that, with respect to the dynamical behavior of the system, several microRNAs form positive feedback loops with p53, typically through direct or indirect inhibition of MDM2.

In this work, we investigate the role of microRNA-mediated positive feedback loops in regulating the performance of p53 stress network. We first developed a mathematical model of a microRNA-p53-MDM2 network that involves three different microRNAs that form positive feedback loops. The core p53-MDM2 model is based on our previously published work [14, 16]. We performed simulations and studied the robustness of p53 oscillations under abrogation of microRNA-mediated positive feedback loops. Furthermore, we adopted bifurcation diagrams in order to explore the system behavior under parametric variability in relationship to cellular noise. To experimentally evaluate our *in silico* predictions, we introduced microRNA inhibitors in a modified breast cancer cell line MCF7, and performed time-lapse microscopy tracking single-cell p53 dynamics under induced DNA double-stranded breaks. Our experimental quantification, in agreement with modeling analysis, reveal that the three microRNA-mediated positive feedback loops confer different level of control to the robust performance of stress-induced p53 oscillations within a population of cells.

## Results

### MicroRNA-mediated positive feedback loops with p53 stress network

In this work, we seek to elucidate the role of miRNAs in the regulation of the p53 oscillation elicited by the stress signal of cellular DNA damage. Among the signaling regulations of p53 induced by miRNAs, we focus on feedback pathways. Intriguingly, three groups of miRNAs that are identified to be a part of feedback regulations of p53, form positive feedback loops with the p53 pathway. The three microRNA-mediated positive feedback networks and the associated molecular interactions are described as follows.

**Fig 1:**
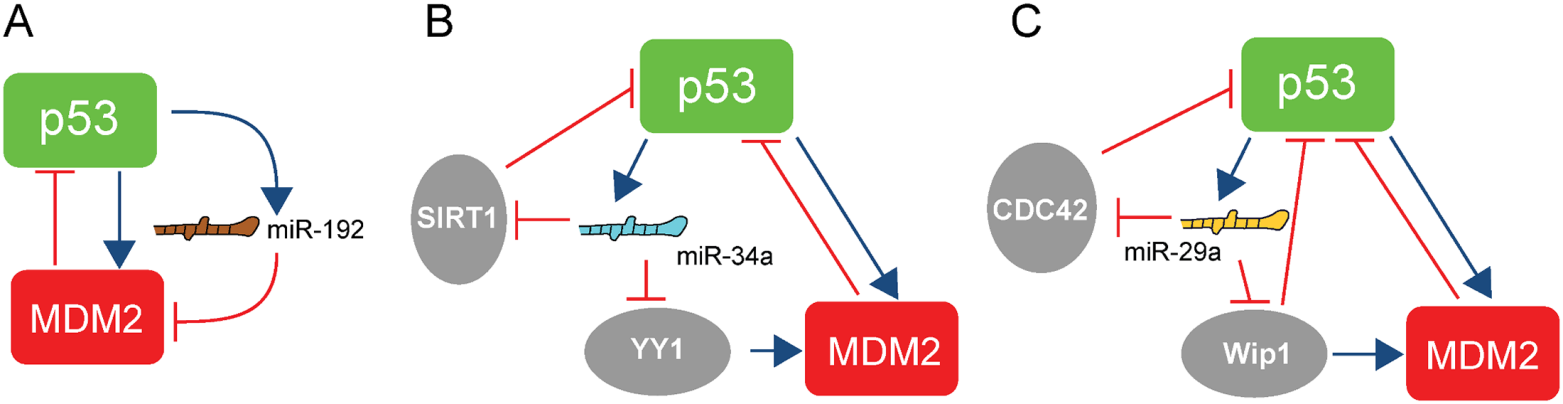
Selected feedback-mediating microRNAs involved in the p53-MDM2 pathway. **(a)** The positive feedback loop is formed by miR-192, MDM2 and p53. **(b)** The positive feedback loop is formed by miR-34a and p53, mediated by SIRT1 or YY1 plus MDM2. **(c)** The positive feedback loop is formed by miR-29a and p53, mediated by CDC42, Wip1, or Wip1 plus MDM2. Note that blue arrows represent activation while the red lines with a hammerhead represent inhibition. Note that the inhibition and activation signs here indicate the regulatory role of a regulator on its target. That is, if the regulation is of an activating (or inhibitory) nature, we use an activation (or inhibition) sign.

The miR-192 family, including miR-192, miR-194 and miR-215 [23], is directly correlated with p53 protein upregulation [24] and overexpression of these microRNAs elicited dramatic down-regulation of MDM2 at protein and mRNA levels. These findings indicate that, on top of the core autoregulation of p53 through MDM2, there is a microRNA-mediated autoregulatory loop of p53, where miR-192 is activated by p53 [25] and in turn inhibits the antagonizing effect of MDM2 [24]. This autoregulatory loop is a positive feedback loop with the feature of double-negative regulation (Fig 1a).

The miR-34 family, including miR-34a, miR-34b, and miR-34c, is upregulated by p53 [26, 27]. Two positive feedback loops between p53 and miR-34a have been reported (Fig 1b). The first loop is through the regulation by the protein named silent information regulator (SIRT1). Specifically, miR-34a inhibits SIRT1 mRNA translation [26, 27]. In irradiated cells the SIRT1 protein acts as an antagonist of the post-translational modification of p53 and thus repressing the transcriptional activity of the p53 protein [28]. The second feedback loop is mediated by Yin Yang 1 (YY1), a ubiquitous transcription factor that negatively regulates p53, and is directly repressed by miR-34a [29]. The YY1 protein can enhance the degradation of p53 promoted by MDM2 [30], thereby closing a feedback loop composed of p53, miR-34a, YY1 and MDM2. Both of the two regulatory loops are positive feedback with double-negative regulations (Fig 1b).

The miR-29 family, including miR-29a, miR-29b, and miR-29c, is upregulated by p53 [31]. The miR-29 family members in turn can enhance p53 activity. For instance, all three miR-29 family members can successfully elevate the phosphorylation level of p53 by repression of Wip1 [31], a phosphatase of p53 [13]. In addition, miR-29 microRNAs directly suppress CDC42 [32], a Rho family CTPase, which directly inhibits the protein activity of p53. Intriguingly, these feedback regulatory pathways are again positive feedback loops in the form of double negative feedback (Fig 1c), and they are closely interlinked with the core p53-MDM2 autoregulation in that Wip1 upregulates MDM2 via inhibiting its degradation [33] (Fig 1c).

### Single-cell model of the microRNA-p53-MDM2 network and cellular variability analysis

**Fig 2:**
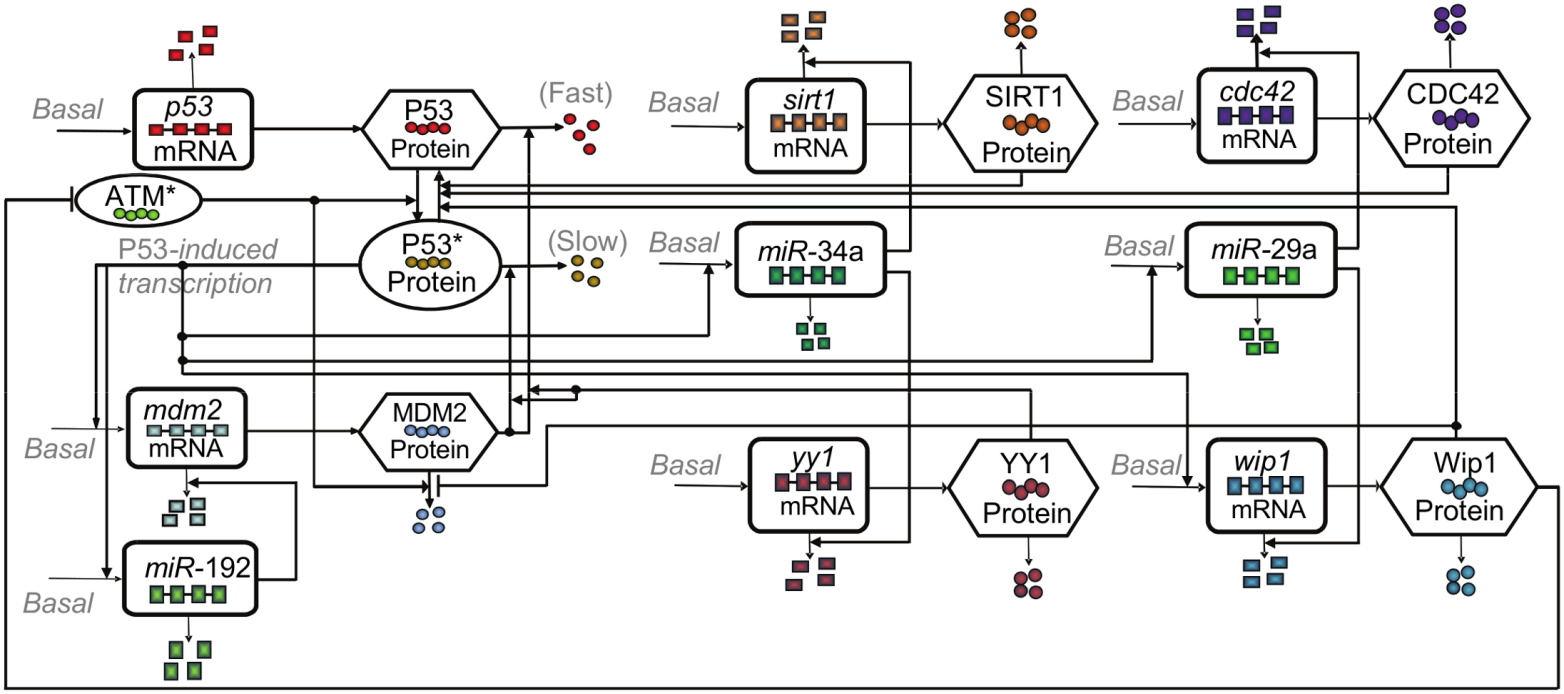
Diagram of the p53-MDM2 oscillator under the regulation of positive feedbacks via microRNA-192, -34a and -29a. p53 is translated from p53 mRNA and remains inactive. Phosphorylated by ATM*, p53 becomes active (p53*), and able to transcribe mdm2 mRNA. MDM2 protein, translated from mdm2 mRNA, promotes a fast degradation of p53 and a slow degradation of p53*. In addition to a basal self-degradation, MDM2 is degraded by a mechanism stimulated by ATM*. The three microRNAs, miR-192, miR-34a and miR-29a, are induced by p53*, and inhibit the mRNAs of mdm2, cdc42, wip1, sirt1 and yy1, whose protein products further regulate p53* and MDM2. Specifically, the microRNA binds with its target mRNA molecule with high affinity, forming a microRNA-mRNA complex, and subsequently dispose the complex into a degradation machinery. In other words, the microRNAs in our model are assumed to enhance the degradation of their mRNA target by complexation and subsequent disposal. In particular, CDC42, Wip1 and SIRT1 proteins deactivate p53 directly, while YY1 enhances the MDM2-dependent degradation of p53 and p53* proteins. In addition, Wip1 protein inhibits the degradation of MDM2 protein. The wip1 mRNA is also induced by p53*, whose protein product inhibits active ATM, forming a second negative feedback loop.

**Fig 3:**
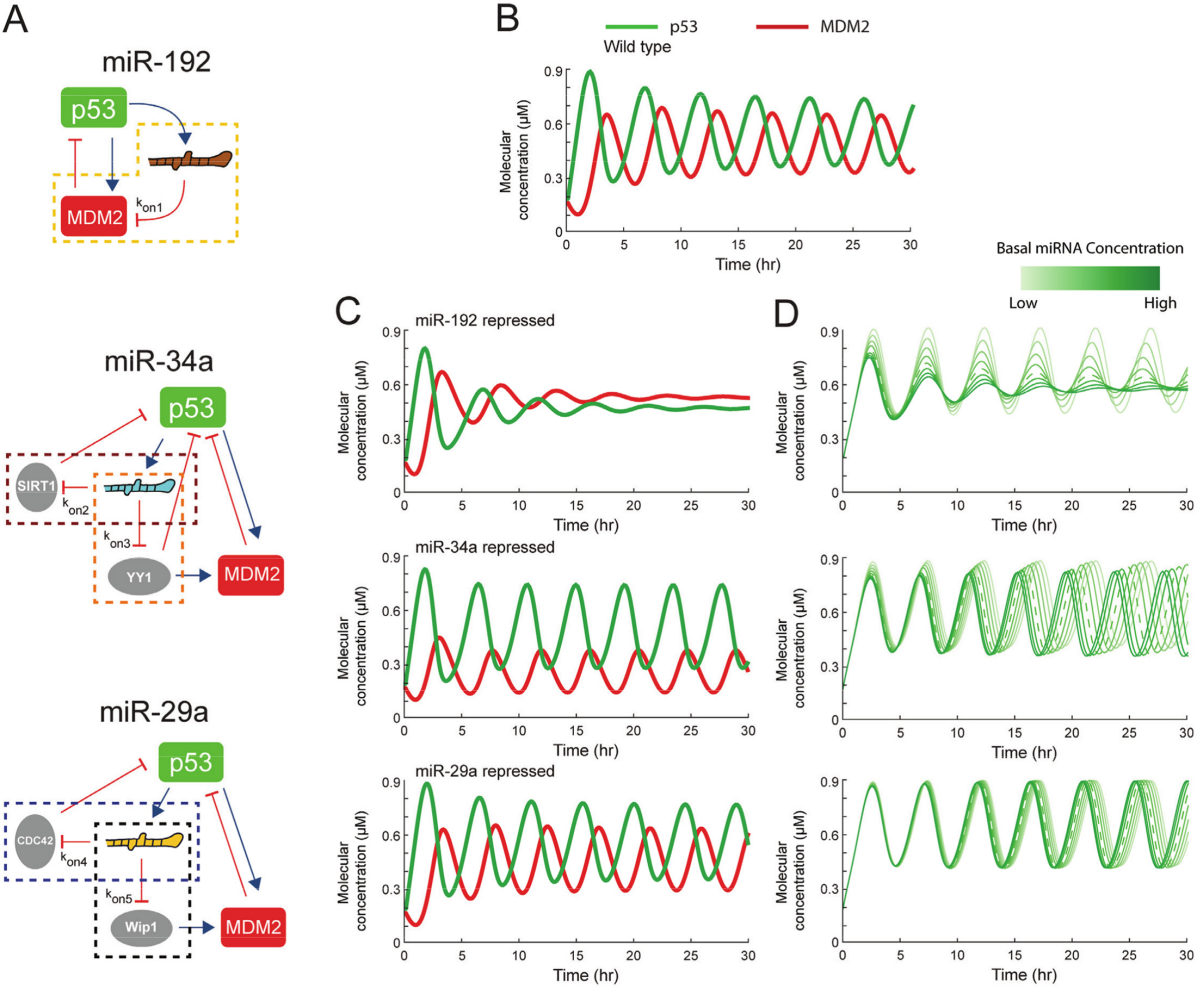
Simulations of the time courses of p53 data shows association rates sensitive to variation. **(a)** Simulations are performed using the ODE model of microRNA-p53-MDM2 network under constant ATM activation. Each microRNA is modeled by accounting for the mediating microRNA component and its associated target proteins. The association rates between the microRNAs (miR 192, 29a and 34a) and target mRNAs (*k*_on1~5_) in this in silico model were varied one at a time to predict its subsequent effect on the oscillatory p53 expression. **(b)** Expression levels of p53 (green) and MDM2 (red) as calculated by the simulation, with a nominal association rate value of 25 for *k*_on1~5_ (top). **(c)** The simulation was repeated with nominal inhibition of miR-192, miR-34a and miR-24a. **(d)**The simulation was repeated at varying level of inhibitor of miR-192, miR-34a and miR-24a by changing the basal induction rate of the microRNAs. The color scale from light green to dark green represents the response with a basal induction rate of microRNAs changing from zero to the respective nominal value, represented by the dotted line, and further above the nominal value.

We first developed a mass-action model that accounts for the core p53-MDM2 autoregulatory network [14, 16] coupled with all the three families of microRNA-based positive feedback loops at single-cell level (Fig 2). Each microRNA is modeled by accounting for the mediating microRNA component and its associated target proteins (i.e. SIRT1 and YY1 for miR-34a, and CDC42 and Wip1 for miR-29a) (Fig 3a). We assume that the microRNA binds quickly with its target mRNA molecule and dispose the microRNA-mRNA complex into degradation [34]. The active form of ATM, a protein kinase that detects DNA damage, is induced by transient DNA damage signal, following the mathematical formula used in Batchelor et al [35]. The assumptions on the interactions among p53, MDM2, microRNAs and intermediate proteins, as well as the ordinary differential equations and parameters of the deterministic single-cell model are included in the Supporting Information (S1 Text, S1 and S2 Tables). Note that our model includes a second negative feedback loop formed by ATM, p53 and Wip1 (Fig 2 and S1 Text), a network structure proposed in previous experimental and computational studies [35, 36]. As shown in a recent study of NF-kB, another oscillatory transcription factor, a longer negative feedback loop in addition to the core faster negative feedback could provide further system properties, such as better tracking of duration of input signal as well as potential induction of damped oscillations [37]. These behaviors potentially allow for more sophisticated signal coding and processing patterns in cellular stress response than that could be achieved by single negative feedback structure.

A simulation of the deterministic model of microRNA-p53-MDM2 network at wild-type condition shows that this system yields oscillations of p53 and MDM2 with period of approximately 5 hours under constant DNA damage stimulus (Fig 3b), equivalent to single-cell response to γ-irradiation or radiomimetic drug observed in our in-house experiments and published experiments [6, 7, 38]. We next probed the behavior of p53 in response to DNA damage and the associated role of the microRNAs. More specifically we introduced to the mathematical model inhibitors that modulate the three microRNAs by complexation reactions. We assume that the microRNAs are repressed by ~6-fold after addition of the inhibitors. Simulations of the deterministic model show that, when miR-192 is inhibited, the stress-induced oscillation of p53 is abolished, but when miR-34a or miR-29a is inhibited the oscillation of p53 persists (Fig 3c). These simulations indicate that at single-cell level the p53 oscillatory behavior is more sensitive to the down-regulation of miR-192 than miR-34a and miR-29a. In light of the deterministic simulation results and considering that a prominent feature of the dynamics of a cell population is the cell-to-cell variability, we decided to investigate further the single-cell system behavior under cellular noise.

The heterogeneity in a cell population has been widely observed in the experiments of the stress response of p53 and other cellular processes [6, 7, 39]. Cellular noise, broadly defined as stochastic fluctuations of molecular processes within and between cells, can be divided into intrinsic and extrinsic noise [40, 41]. Intrinsic noise refers to random deviation of the molecular processes from their average deterministic kinetics within a cell, mostly due to probabilistic biochemical reactions associated with low copy numbers. Several attempts have been made to use stochastic models to study the noisy single-cell p53 dynamics under the influence of intrinsic noise due to low copy number of reactants [42, 43]. Nevertheless, the high molecule numbers measured in the p53 network (10^4^−10^5^) [14, 44] suggest that the role played by intrinsic noise may not be critical especially in the variable induction of oscillatory and non-oscillatory phenotypes in single cells [6], as intrinsic noise mostly just results in irregular profiles of a trajectory with high copy numbers [42]. Indeed, a previous study shows that oscillation produced by limit cycle seems to be very robust under intrinsic stochasticity, where the simulated stochastic oscillations persist when the maximum molecule numbers are in the order of hundreds [45]. On the other hand, extrinsic noise generally dominates cellular stochasticity, especially in eukaryotic systems [46–48], and arises from global factors that impact cell-to-cell variation [49]. Therefore, in this study we focus on analyzing the impact of extrinsic noise on the sustainability of p53 oscillation at single cell level. To this end, deterministic single-cell model with varying model parameters can be used to compute the impact of extrinsic noise [50–52].

In our experimental setup, the perturbations are performed using the inherently “noisy” transient transfections of microRNA inhibitors, resulting to variable down-regulation levels between cells. To probe the effect of microRNA inhibitor copy-number variability we first performed parametric simulations for a wide range of inhibitor concentrations. As illustrated in Fig 3d, the effect of the miR-192 inhibitor is more prominent, leading to gradual collapse of the oscillating p53 behavior at high concentrations.

**Fig 4:**
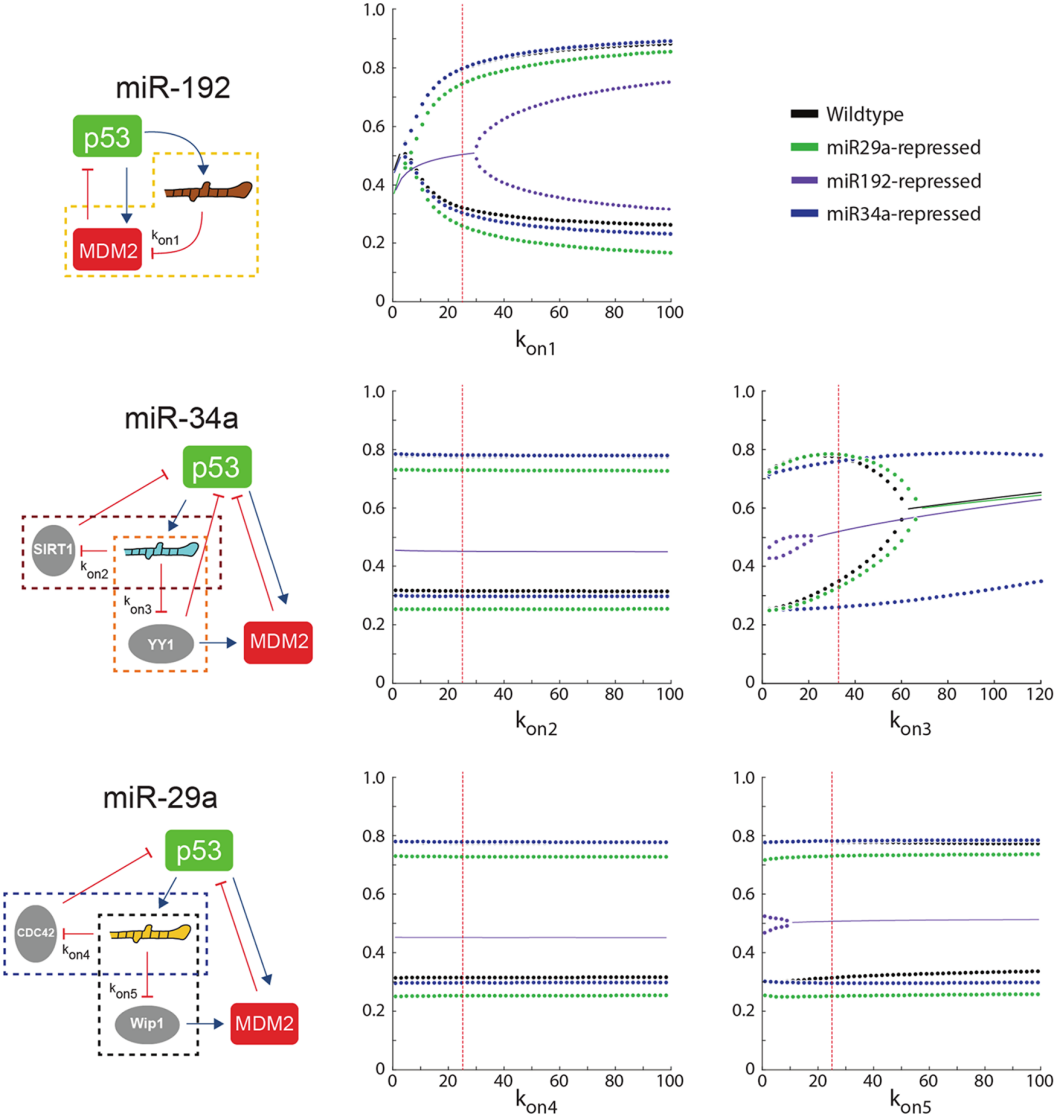
Bifurcation diagrams of the DNA double-strand-break triggered steady-state p53 response versus the association rates between the three microRNAs and their five target mRNAs (*k*_on1_, *k*_on2_, *k*_on3_, *k*_on4_, *k*_on5_) embedded in the positive feedback loops, under the wild-type (black), miR-192 repressed (purple), miR-34a repressed (green), and miR-29a repressed (blue) conditions. Paired dots represent the bounds of p53 oscillation amplitude and the solid line represents stationary steady state. The red vertical line indicates the nominal parameter value. The inhibition of miR-192 leads to shrunk oscillating region or entirely non-oscillating region over varying parameter ranges. The inhibition of miR-34a and miR-29a only mildly affects the system behavior compared to the wild-type condition.

To further investigate the effect of microRNA abrogation under cellular extrinsic noise we performed bifurcation analysis. We assayed model parameters along the microRNA-mediated positive feedback loops that directly affect the transduction of the microRNA perturbation through the network. In particular, we probed 14 parameters under wild-type and the three microRNA-repressed conditions (S3 Table). First, we calculated the bifurcation diagrams of the steady-state p53 concentration versus the 5 association rates between the microRNAs and their target mRNAs for the wild-type case as well as the three perturbed cases with microRNA inhibitors (Fig 4). The paired dots represent the bounds of p53 oscillation amplitude at steady state and the solid line represents stationary steady state. According to Fig 4, the miR-192 inhibitor either reduces the range of the oscillatory p53 response, or abolishes p53 oscillation, across different affinities of miR-192 to its target. Note that the p53 response at the nominal parameter set is still within oscillatory region when miR-34a and miR-29a are inhibited, while it falls out of the oscillatory region when miR-192 is repressed. This is consistent with the persistent oscillations in the former two cases and the stationary steady state in the latter case, as shown in Fig 3b. The bifurcation diagrams versus the rest 9 parameters are shown in S1 Fig.

It is noteworthy that the experiments by Geva-Zatorsky *et al* showed that the amplitude of p53 in individual cells is highly variable with a variation up to ~70%. There have been attempts to implement the high variation of p53’s amplitude by theoretical modeling. For instance, Jolma *et al* assumed that certain rate parameters were allowed to vary randomly and rapidly within a certain range to achieve the variable p53 amplitude [53]. Such method essentially implements the extrinsic noise computationally as explained above. In our model, the variable p53 amplitude can also be induced by extrinsic noise via allowing variations in parameters, whereby the impact of varying parameters on p53 amplitude is demonstrated by the bifurcation plots (Fig 4 and S1 Fig). For instance, varying the value of *k*_on1_ alone between [10, 40] achieves ~65% variation of the p53 amplitude (see the wild-type case in the plot with respect to *k*_on1_ in Fig 4). Also, varying the value of *k*_w_ alone between [1, 3] achieves ~53% variation of the p53 amplitude (see the wild-type case in the plot with respect to *k*_w_ in S1 Fig). Therefore, significant variation in p53 amplitude in our model is attainable by assuming considerable extrinsic noise in the model parameters.

The bifurcation diagrams with respect to each of the 14 parameters embedded in the microRNA-based positive feedback loops parameters (S3 Table) reveal that for 8 out of the 14 parameters the repression of miR-192 leads to the smallest regions of oscillation compared to those of miR-34a and miR-29, while for the other 6 parameters the repression of miR-192 completely abolishes the p53 oscillation over the varying ranges (Fig 4 and S1 Fig). These plots indicate that the non-oscillating phenotype of an individual cell can be yielded when certain parameter, due to extrinsic fluctuation, is pushed out of the bounds of its oscillatory regime, thus providing a plausible mechanism underlying the observed heterogeneous behavior of p53 in a cell population. Moreover, if the region of p53 oscillation significantly shrinks, it is more likely for the oscillatory behavior to be ruined by extrinsic noise, and thus the probability of observing non-oscillating single-cell phonotype in a stochastic population should increase.

To further quantify the impact of different types of microRNAs on regulating the p53 network, we measured the system’s robustness performance, which is the capability to maintain the oscillatory behavior of p53 in response to DNA damage (S2 Text). A large robustness index defined in S2 Text predicts that the system’s probability of sustaining stable oscillation under stochastic perturbations is relatively high. As a result, for the model under a particular condition the value of its robustness index is positively correlated with the fraction of oscillating cells in a population. The robustness indices confirm that when miR-192 is repressed the oscillatory phenotype is the least robust among the three microRNAs (S4 Table). Based on the model predictions, we infer that the inhibition of miR-192-mediated positive feedback loop would lead to the highest probability of non-oscillating cells across a population due to extrinsic noise.

As a summary, the theoretical modeling and analysis show that the robust performance of the p53 stress network is subject to the control of specific microRNA-feedback regulation.

### Experimental abrogation of microRNA-mediated positive feedback loops in MCF7 cells

**Fig 5:**
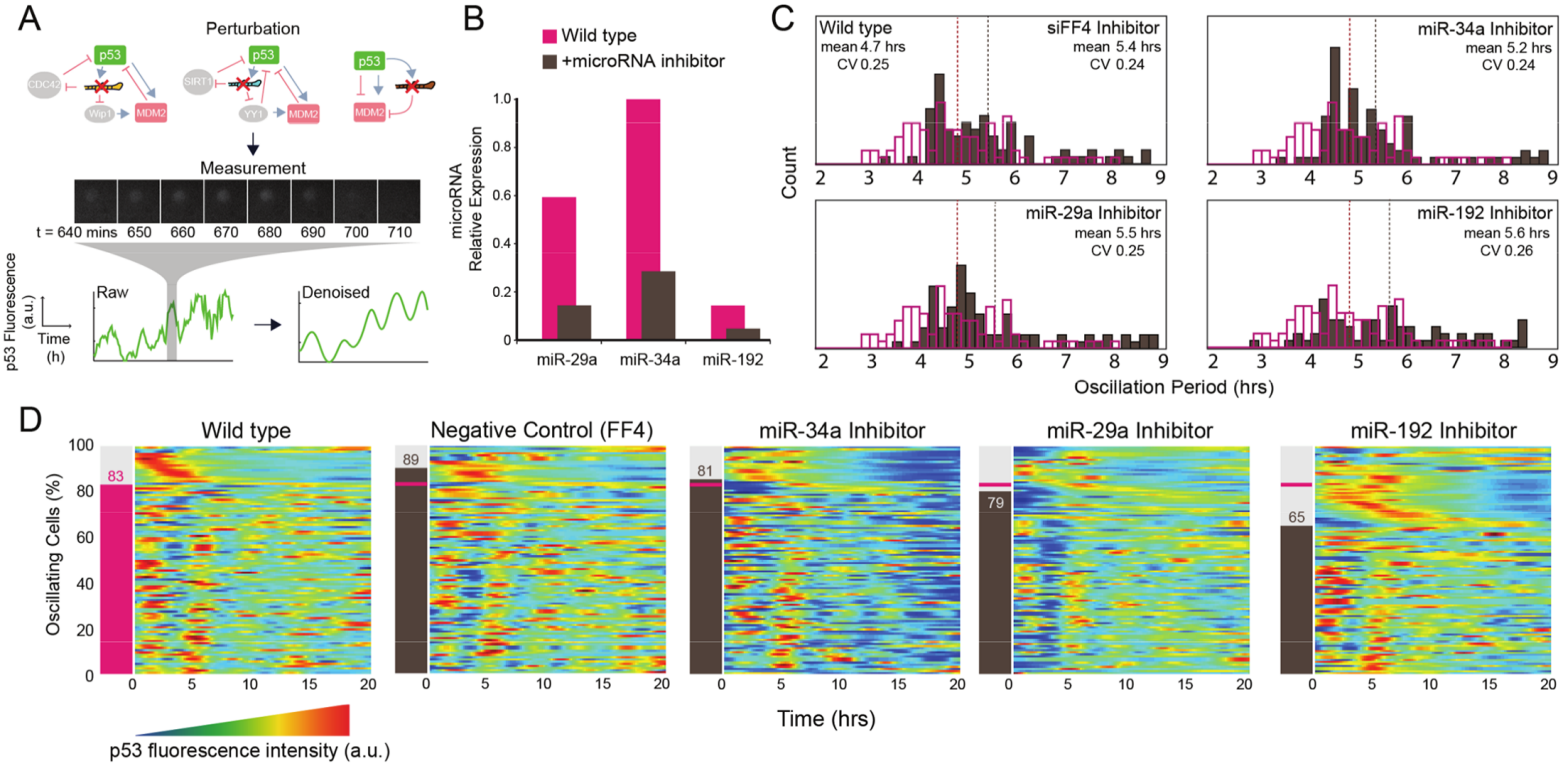
Experimental perturbation of p53-associated microRNA affects oscillatory behavior. **(a)** To examine the role of microRNA 29a, 34a and 192 in the stress-induced p53 oscillations, expression of three endogenous microRNAs known to be associated with p53 activation pathway is targeted using microRNA inhibitor. Fluorescence is captured every 10 minutes for 20 hours following transfection. Single cells are identified and their intensity is recorded to create a time series data for each cell. **(b)** Quantitative PCR measurements of microRNA levels before and after treatment with the microRNA inhibitor. **(c)** Period distribution of p53 oscillation before and after treatment with various microRNA inhibitor. Magenta bars represent period of p53 oscillations observed in wild type population, and brown bars represent the same measurement from the population treated with the specified microRNA inhibitor. **(d)** Heatmaps showing single cell tracks of p53 fluorescence measurement after various microRNA inhibitor treatment. Each graph has 100 rows, each row representing a single cell tracking data for 20 hours. Data shown has been reorganized to reflect the results from the classification process to identify cells with oscillatory p53 expression. Number on the left shows the number of cells identified as oscillating for each group, where the magenta bar indicates the wild-type level.

To experimentally probe the effect of microRNA abrogation on the p53-MDM2 oscillator we used a breast cancer cell line MCF7 [38] that contains a stably integrated fluorescent reporter Venus fused to the cDNA of p53 under the expression of the metallothionein promoter (Fig 5a). To down-regulate the desired microRNAs we introduced to MCF7 cells synthesized single-stranded RNA molecules that are complementary to the mature microRNA sequence.

Prior to performing the microRNA perturbation experiments, we verified the expression of the three selected microRNAs using quantitative PCR (qPCR). We confirmed the expression of miR-29a, miR-192, and miR-34a as well as efficacy of their inhibitors (Fig 5b); results from qPCR show between 70% to 80% down-regulation for each microRNA targeted.

We then performed two independent time-lapse experiments to test the following five conditions: wild type (WT) untransfected cells, negative control with transfection using a synthetic microRNA that does not target the p53-mdm2 core, and the three selected microRNA inhibitors. For the negative control case we transfected the MCF7 cells with a synthetic microRNA (FF4) [54] that does not interfere with the p53-MDM2 core.

The oscillations of p53 protein after addition of the microRNA inhibitors and neocarzinostatin (NCS) [38] were captured using time-lapse microscopy. The details of the preparation of the wild type and microRNA-perturbed MCF7 cells and the subsequent time-lapse microscopy are described in the Materials and Methods Section. The image data acquired from the time-lapse microscopy were processed using ImageJ [55] and MATLAB. First, the fluorescence signal of the nuclear-localized p53 cells in the image stack was tracked and the average fluorescent intensity of p53 in individual cells was recorded for each cell at 10-minute intervals for 20 hours.

To evaluate the impact of the microRNA perturbations on the stress-induced p53 oscillations, we analyzed one hundred single-cell trajectories of p53 fluorescence profiles, after artificially induced DNA damage. The raw trajectory data was denoised using the stationary wavelet transform (SWT) in MATLAB [56] (S2 Fig). After smoothing out the raw signal intensities to reduce noise, we classified each denoised p53 trajectory data by extracting relevant parameters of the intensity profile to help us determine whether the observed fluorescence profile possesses qualities consistent with typical p53 oscillation or not (Materials and Methods).

Specifically, we located relevant peaks of the time-series data and recorded their locations to identify cells that have abnormally long or short periods. If two consecutive peaks occur within 50 minutes or at least 12 hours apart, the cell was eliminated from being classified as oscillatory. For a better look at the overall trend of the dynamic fluorescence signal, we also calculated the instantaneous slope of the fluorescence profile and the frequency of the time that it is below zero during the 20 hour span. If the slope was zero more than 70% of the 20 hour period, we exclude the cell from being classified oscillating and vice versa. The entire classification process was automated in MATLAB, and the classified cell-population results of the duplicate experiments under WT, negative control, as well as the three microRNA-inhibited conditions are illustrated in S3–S7 Figs.

After sorting our time-lapse data for individual cells showing oscillation, we found that the targeted microRNA suppression seems to affect the stress-induced p53 oscillation quantitatively in terms of the number of cells with oscillating p53 expression, but have a little qualitative effect on the period or amplitude of the oscillations. Specifically, we found the mean oscillation period among the selected cells to be approximately 5 hours, and this mean period remained stable after suppression of the three targeted microRNAs. As expected, we found that there was non-negligible level of cell-to-cell variability in the oscillation period based on the coefficient of variation. More importantly, we found that the microRNA suppression seems to have little effect on this variability (Fig 5c).

We then calculated the percentage of oscillating cells after each microRNA abrogation. In our time-lapse experiment, fluorescence signal from 100 cells were captured every 10 minutes over a 20-hour time period in 5 different conditions, giving us over 60,000 data points per experiment. To present this data efficiently, we employ heatmaps composed of pixels that each represents a single data point. Each row of the graph represents the p53 signal intensity trajectory of a single-cell, and each column represents a single time point. Color at each pixel is indicative of the relative intensity of the p53 signal. To highlight the differences between the fluorescence profiles of oscillating and non-oscillating cells, we re-organize the time-series heatmaps into two populations and re-order them based on the location of the first observed peak. We found that the cells transfected with the inhibitor of miR-192 show markedly decreased number of p53 oscillating cells comparing to wild type, while the population affected by inhibitors of microRNA 29a and 34a show similar occurrence rate of oscillation as the wild type (Fig 5d). Note that the results from duplicate experiments show the same trend of reduced number of oscillating cells in a population only when miR-192 is repressed, although the absolute value differs (S5 Table).

## Discussion

Here we use an approach of theoretical modeling combined with quantitative experiments to elucidate the role of microRNAs on the cellular performance of oscillatory p53 induced by DNA double-strand breaks. Our results show that the microRNA-mediated positive feedback loops influence the robust manifestation of stress-induced p53 oscillations in stochastic cellular systems. Specifically, the repression of miR-192 led to widespread collapse of the sustained p53 oscillations across a population of variable cells while the repression of miR-34a and miR-29a mildly affected the phenotype under double-stranded DNA damage.

A functional role of microRNAs has been proposed in that they confer robustness to biological processes [57], including cellular differentiation in development or tumorigenesis [58]. Notably, the microRNA-mediated functional network motifs that previously have been discovered to bestow the function of robust maintenance of cell fate are all positive feedbacks, consisting of a transcription factor and a microRNA, either with or without intermediate signaling components [58]. Our findings add new evidence of microRNA-mediated positive feedback loops that function as a mechanism that reinforces the robustness of a system phenotype.

Bifurcation analysis, a method widely used in engineering to evaluate system robustness, provides effective means to delineate the variable behavior of single cells subject to extrinsic noise in parameters. Our bifurcation diagrams of the single-cell model of microRNA-p53-MDM2 network show substantially higher reduction of oscillation regions under the inhibitions of miR-192 comparing to the other two microRNAs. Theoretical studies of the biochemical oscillators arising from a core negative feedback loop plus an additional positive feedback loops have been performed recently [59, 60]. Specifically, a modified Goodwin model consisting of a three-component negative feedback loop interlinked with different positive feedback motifs was studied for the performance of the oscillator with regard to the benefits acquired by the auxiliary positive feedback regulations. This is analogous to our wild-type model, where the core mechanism for the p53 oscillator is the p53 protein-MDM2 mRNA-MDM2 protein negative feedback loop, and it is coupled with positive feedback loops. Besides the advantages that may be gained for the oscillator by positive feedback loops, their results show that a positive feedback loop is the most beneficial for the robust performance of the oscillator if its pathway components have the fastest dynamic, such as the fastest degradation rate. In other words, the finding predicts that a positive feedback loops with faster information transduction on top of the core negative feedback is a more favorable structure to stabilize oscillation. We can apply this finding qualitatively to interpret our model behavior. For the positive feedback regulations in our model, miR-192 directly regulates MDM2, while miR-34a and miR-29a regulate intermediate nodes prior to reaching MDM2, before the MDM2 information is eventually fed back to p53 protein to close the loop. Consequently the miR-192 mediated double-negative feedback loop contains shorter signal-processing path than that of the miR-34a- and miR-29a-mediated feedback loops. Although miR-34-a and miR-29a each also forms a short positive feedback loop together with a mediating protein with the same length as the miR-192-MDM2 feedback loop, the MDM2 protein is degraded at a faster rate upon DNA damage and thus the latter loop would still contribute the most to the system robustness. We therefore infer that among the three groups of microRNAs forming positive feedback loops with p53, miR-192 and its mediated feedback pathway plausibly exert the greatest impact on maintaining the robustness of p53 oscillations in response to DNA damage.

A previous modeling study has proposed the role of a different positive feedback loop in enhancing the robust stability of p53 oscillation [61], whereby the cytosolic MDM2 protein translocates into nucleus to interact with the mRNA of p53 through direct binding and promote the translation of p53 mRNA to close the loop [62, 63]. Such positive feedback is a relatively long and slow loop involving steps of the compartmental trafficking of MDM2 protein from cytosol into nucleus, the protein-mRNA binding between MDM2 protein and p53 mRNA, and the final translation of p53 protein induced by MDM2 protein. The microRNA-mediated positive feedback loop, on the other hand, is more efficient. For instance, the positive feedback mediated by miR-192 is only composed of fast binding of microRNA to MDM2 mRNA and the post-translational degradation of p53 protein promoted by MDM2 protein. The core negative feedback is also a fast and efficient loop containing the post-translational degradation of p53 protein promoted by MDM2 protein. Note that the same type of processes in the positive feedback loops, such as the induction of mRNA or microRNA by p53 and the translation of MDM2 protein, is not enumerated in the above comparison. In addition, we note that translational process is in general much slower than post-translational regulation. In sum, the positive feedback facilitated by MDM2-enhanced translation of p53 occurs in a much lower efficiency than the core p53-MDM2 negative feedback and the microRNA-mediated positive feedback loops. It thus is reasonable to assume that the positive feedback loop through the MDM2-enhanced translation of p53 has relatively weak impact on the p53 oscillation. This probably is the reason why the recent major theoretical studies of the p53 oscillator do not account for the positive feedback loop through the MDM2-enhanced translation of p53 [36, 42, 64–66]. Indeed, if we add an MDM2-dependent translation term into the ordinary differential equation of the p53 mRNA with a translational rate half that of the basal translational rate to approximate the slow processes due to MDM2 translocation and MDM2-p53mRNA interaction, the simulations of the p53 oscillation are very similar to the model without the positive feedback through the MDM2-enhanced translation of p53, indicating that this positive feedback does not have much impact on the p53 oscillation.

In conclusion, our modeling and experimental results provide new evidence on the relationship between microRNAs and p53 function [23] with implications to cancer initiation and progression. Importantly, understanding the mechanisms underlying the abnormal p53 behavior due to microRNA depletion [67] may lead to innovative microRNA-based therapeutics.

## Materials and Methods

### Tissue culture

The MCF7 breast cancer cell line, consisting of the fluorescent protein Venus fused to p53, is the same as previously described [9, 38], a gift from Galit Lahav, Harvard Medical School. Cells were maintained in 95% humidity at 37 degrees Celsius and cultured in RPMI (Invitrogen) media with 10% FBS (Invitrogen), 1% PenStrep (Invitrogen 0.045 units/mL Penicillin, 0.045 units/mL Streptomycin). After the first splitting following resurrection from liquid nitrogen, stably integrated MCF7 cells were maintained at 20 mL volume in petri dishes with 400ug/mL G 418 disulfate salt (400ng/mL, Sigma). In a 12 well plate (Griener), 80,000 MCF7 p53-Venus cells were plated on the afternoon before transfection. The following morning, the cells were treated with Neocarzinostatin (Sigma) and transfected with 3ul JetPRIME (Polyplus) mix with 25nM of the microRNA inhibitors 192, 29a and 34a (Qiagen) according to the manufacture’s protocol. To stimulate the activity of p53 we added the radiomimetic drug Neocarzinostatin (NCS), which induces the particular lesion of DNA double-strand breaks and elicits p53 to oscillate [35, 38]. Following the addition of 0.8 μl NCS per well from 0.5 mg/mL stock (Sigma) and either with or without the transfection of microRNA inhibitors we commenced a time-lapse microscopy at 37 degrees Celsius with humidified 5% CO2.

### Fluorescence microscopy

Images were collected every 10 minutes for the bright field and fluorescent intensity of p53-Venus using a Hamamatsu camera attached to the Olympus IX81 microscope at 10x magnification. The time lapse ran for 24 hours and used exposure times of 10ms for Bright Field and 500ms for YFP. We chose three positions for each well, ensuring that the imaging field did not overlap between positions. The filter used for capturing Venus fluorescence is excitation ET500/20x and emission ET535/30m (Chroma). Cells were incubated within the microscope at 37 degrees Celsius with approximately 5% CO2.

### Image processing and data analysis

The image stacks were first processed by an ImageJ plug-in CGE to measure and track the average intensity of nuclear p53 in single cells [55]. For a specific cellular condition, we tracked an average of 33–34 cells within each of the three locations, and a total number of 100 cells, for 20 hrs (see the tracked cell trajectories in S6 Table). The raw time trajectories of p53 intensity for 100 cells then underwent a de-noising step implemented by the Stationary Wavelet Transform De-Noising 1-D Tool of MATLAB to remove the high-frequency noise and extract the low-frequency p53 oscillation [56] (S2 Fig). Finally, each trajectory was subject to a classification algorithm for the purpose of determining its phenotype, oscillating or non-oscillating. Specifically, to obtain additional characteristics from the resulting p53 trajectories, instantaneous slope at each point was calculated using MATLAB code *diff*, and the peaks were detected using MATLAB code *mspeaks*. Instantaneous slope of each trajectory, along with the locations of its peaks, were used to determine the oscillating and non-oscillating phenotypes of individual cells. The single-cell trajectories were classified as non-oscillating (S3–S7 Figs) if: (a) less than 3 peaks were detected during the time-lapse, (b) there were 2 consecutive peaks that occur within 5 time units (100 min), (c) there were 2 consecutive peaks that occur more than 70 time units (1400 min) apart, and (d) the slope of the trajectory was negative (or positive) more than 75% of the time. Otherwise, the trajectory was classified as oscillating.

## Supporting Information

S1 Text Modeling deterministic single-cell microRNA-p53-MDM2 network. (PDF)

S2 Text Robustness analysis of the single-cell model of microRNA-p53-MDM2 network. (PDF)

S1 Table List of the 22 molecular species used in the deterministic model. (PDF)

S2 Table List of the 90 parameters used in the deterministic model. (PDF)

S3 Table List of the fourteen parameters along the microRNA-based positive feedback loops that are subject to bifurcation analysis in S2 Text. (PDF)

S4 Table The Robustness Index (RI) of the microRNA-p53-MDM2 system with respect to the fourteen selected parameters under the wild-type and microRNA-repressed conditions. The abbreviation “*wrt*” denotes “with respect to”.
(PDF)

S5 Table Percentage of cells that oscillate following treatment with microRNA inhibitor in duplicate experiments. (PDF)

S6 Table Time trajectories of the tracked p53 fluorescence intensity in single cells acquired by the repeated time-lapse microscopy experiments. (XLSX)

S1 Fig Bifurcation diagrams of the DNA double-strand-break triggered steady-state p53 response versus 9 parameters (*ε*_miRNA1_, *ε*_miRNA2_, *ε*_miRNA3_, *k*_da1_, *k*_da2_, *k*_da3_, *k*_w1_, *v*_p53_ and *k*_yy1_) embedded in the positive feedback loops, under wild-type (black), miR-192-repressed (purple), miR-34a-repressed (blue), and miR-29a-repressed (green) conditions. A paired dots represent the bounds of p53 oscillation amplitude and the solid line represents stationary steady state. The red vertical line indicates the nominal parameter value. Again, the inhibition of miR-192 leads to shrunk oscillating region or completely non-oscillating region over varying parameter ranges, while the inhibition of miR-34a and miR-29a mildly affects the systems behavior.
(PDF)

S2 Fig MATLAB GUI (graphical user interface) of the Stationary Wavelet Transform 1-D tool. Through this step, the raw signal of p53 trajectory is decomposed into a smooth denoised signal and high-frequency residuals.
(PDF)

S3 Fig Detection of oscillation in wild type p53 fluorescence trajectory in duplicate experiments. After denoising, peaks and instantaneous slope of each single-cell tracking data obtained before determining whether the observed p53 fluorescence expression was considered oscillatory. Each trajectory is tested for following conditions to determine whether it is oscillatory: 1) less than 3 peaks were detected during the time-lapse, 2) there were 2 consecutive peaks that occur within 5 time units (100 minutes), 3) there were 2 consecutive peaks that occur more than 70 time units (1400 minutes) apart, and 4) the slope of the trajectory was negative (or positive) more than 75% of the time. Trajectories of cells that exhibited criteria 1, 2 and 3 are considered non-oscillating and are shaded in grey. Cells that fall into category 4 are shaded in pink. Number below each graph indicates the ratio of time in which the slope of trajectory is negative.
(PDF)

S4 Fig Detection of oscillation in FF4-injected p53 fluorescence trajectory in duplicate experiments. See S3 Fig for a detailed description.
(PDF)

S5 Fig Detection of oscillation in mir29a-inhibited p53 fluorescence trajectory in duplicate experiments. See S3 Fig for a detailed description.
(PDF)

S6 Fig Detection of oscillation in mir34a-inhibited p53 fluorescence trajectory in duplicate experiments. See S3 Fig for a detailed description.
(PDF)

S7 Fig Detection of oscillation in mir192-inhibited p53 fluorescence trajectory in duplicate experiments. See S3 Fig for a detailed description.
(PDF)
