# Supplementary material for: MiR-192-Mediated Positive Feedback Loop Controls the Robustness of Stress-Induced p53 Oscillations in Breast Cancer Cells
Source: PLoS Comput Biol. 2015 Dec 7;11(12):e1004653. doi: 10.1371/journal.pcbi.1004653 (PMC4671655; doi:10.1371/journal.pcbi.1004653)
Supplement: S1 Text — (PDF) [file pcbi.1004653.s001.pdf]

S1 Text. Modeling deterministic single-cell microRNA-p53-MDM2 network

The schematic diagram of the microRNA-p53-MDM2 oscillator is illustrated in **Figure 2**. The interaction scheme among the mRNAs and the proteins of p53 and MDM2 is the same as our previous model [1], except that we have removed the explicit time delays, as recent modeling work has shown that the p53 negative feedback with the intermediate steps of mRNA in addition to the instability endowed by a positive feedback loop could properly provide the sustainability of the p53 oscillation [2]. In brief, p53 protein is translated from *p53* mRNA and is inactive for transactivation of its targets unless phosphorylated by ATM^*, the active form of ATM that senses and transmits DNA damage signal. In its active state, $p53^*$ transcribes *Mdm2* mRNA, in addition to the p53-independent constant basal transcription rate. MDM2 protein promotes a fast degradation of p53 and a slow degradation of $p53^*$. MDM2 has a basal degradation rate, and an ATM^* promoted degradation. The interconnections with the three microRNAs miR192, miR34a and miR29a shown in Figure 1 of the main paper have been integrated. The detailed regulations and the corresponding mathematical formulations are explained below.

In the past few years, a new perspective has been put forth that the p53 pulses are a series of independent pulses repeatedly generated by an excitable system and not the result of a limit-cycle oscillator [3, 4]. Notably, under constant input of ATM, p53 still presents sustained pulses, but not single pulse as predicted by a deterministic excitable system [4]. Though elicited by noise, an excitable system may fire pulses repeatedly with random timing. On the contrary, the stress-induced p53 pulses exhibit rather stable periodicity in experiments, showing a feature of plausible noisy limit-cycle oscillator. Therefore, in this study we assume that the deterministic single-cell p53 response to stress-induced DNA double-strand breaks is based on our previous theory of nonlinear oscillator. In addition, although several other negative feedback regulations of p53 have also been suggested, there exists a consensus in the literature that the p53-MDM2 autoregulatory loop is the essential architecture for the p53 network and its periodic activity. Due to this reason, and the prevailing reductionism used in mathematical modeling, our model takes into account of the core negative-feedback structure of the autoregulation between p53 and MDM2, as well as a secondary ATM-p53-Wip1 negative feedback (see the main text and details in item (G) below). Following the same reductionism rationale, the positive feedback induced by ROR-α [2] is not included in this model, which allows us to focus on the effect due to the positive feedback loops mediated by microRNAs.

The ODEs used in the paper to model the microRNA-p53-MDM2 network of **Figure 2** are:

(1) $\frac{d[p53]}{dt} = s_{p53} - \delta_{p53}[p53]$

(2) $\frac{d[mdm2]}{dt} = s_{mdm2} + \varepsilon_{Mdm2}\frac{[\mathrm{P53}^*]^4}{[\mathrm{P53}^*]^4+K_M{}^4} - \delta_{mdm2}[mdm2] - k_{on1}[mdm2]*[miRNA1]$
$+k_{off1}[mdm2-miRNA1]$

(3) $\frac{d[\mathrm{P53}]}{dt} = r_{\mathrm{P53}}[p53] - \mu_{\mathrm{P53}}[\mathrm{P53}] - \upsilon_{\mathrm{P53}}[\mathrm{MDM2}]\frac{[\mathrm{P53}]}{[\mathrm{P53}]+K_d} * k_{yy1} * \frac{[\mathrm{YY1}]}{[\mathrm{YY1}]+K_y} + k_{rp}[\mathrm{P53}^*] -$
$k_{fp}[\mathrm{ATM}^*]\frac{[\mathrm{P53}]}{[\mathrm{P53}]+K_p} + k_{da1}[\mathrm{SIRT1}]\frac{[\mathrm{P53}^*]}{[\mathrm{P53}^*]+K_{da1}} + k_{da2}[\mathrm{CDC42}]\frac{[\mathrm{P53}^*]}{[\mathrm{P53}^*]+K_{da2}} + k_{da3}[\mathrm{WIP1}]\frac{[\mathrm{P53}^*]}{[\mathrm{P53}^*]+K_{da3}}$

(4) $\frac{d[\mathrm{P53^*}]}{dt} = k_{fp}[\mathrm{ATM^*}]\frac{[\mathrm{P53}]}{[\mathrm{P53}]+K_p} - k_{rp}[\mathrm{P53^*}] - (v_{\mathrm{P53}}/k_v)[\mathrm{MDM2}]\frac{[\mathrm{P53^*}]}{[\mathrm{P53^*}]+K_d^*} * k_{yy1} * \frac{[\mathrm{YY1}]}{[\mathrm{YY1}]+K_y} -$

$k_{da1}[\mathrm{SIRT1}]\frac{[\mathrm{P53^*}]}{[\mathrm{P53^*}]+K_{da1}} - k_{da2}[\mathrm{CDC42}]\frac{[\mathrm{P53^*}]}{[\mathrm{P53^*}]+K_{da2}} - k_{da3}[\mathrm{WIP1}]\frac{[\mathrm{P53^*}]}{[\mathrm{P53^*}]+K_{da3}}$

(5) $\frac{d[\mathrm{MDM2}]}{dt} = r_{\mathrm{MDM2}}[mdm2] - \{\mu_{\mathrm{MDM2}} + (\nu_{\mathrm{MDM2}} - \mu_{\mathrm{MDM2}})\frac{[\mathrm{ATM^*}]}{[\mathrm{ATM^*}]+K_a}k_w\frac{K_w}{[\mathrm{WIP1}]+K_w}\}[\mathrm{MDM2}]$

(6) $\frac{d[miRNA1]}{dt} = s_{miRNA1} + \varepsilon_{miRNA1}\frac{[\mathrm{P53^*}]^4}{[\mathrm{P53^*}]^4+K_1{}^4} - \delta_{miRNA1}[miRNA1] - k_{on1}[mdm2] * [miRNA1] + k_{off1}$

$[Mdm2 - miRNA1]$

(7) $\frac{d[mdm2-miRNA1]}{dt} = k_{on1}[mdm2] * [miRNA1] - k_{off1}[mdm2 - miRNA1] - \delta_{M-mi1}[mdm2 - miRNA1]$

(8) $\frac{d[miRNA2]}{dt} = s_{miRNA2} + \varepsilon_{miRNA2}\frac{[\mathrm{P53^*}]^4}{[\mathrm{P53^*}]^4+K_2{}^4} - \delta_{miRNA2}[miRNA2] - k_{on2}[sirt1] * [miRNA2] +$

$k_{off2}[sirt1 - miRNA2] - k_{on3}[yy1] * [miRNA2] + k_{off3}[yy1 - miRNA2]$

(9) $\frac{d[sirt1-miRNA2]}{dt} = k_{on2}[sirt1] * [miRNA2] - k_{off2}[sirt1 - miRNA2] - \delta_{s-mi2}[sirt1 - miRNA2]$

(10) $\frac{d[yy1-miRNA2]}{dt} = k_{on3}[yy1] * [miRNA2] - k_{off3}[yy1 - miRNA2] - \delta_{y-mi2}[yy1 - miRNA2]$

(11) $\frac{d[sirt1]}{dt} = s_{sirt1} - \delta_{sirt1}[sirt1] - k_{on2}[sirt1] * [miRNA2] + k_{off2}[sirt1 - miRNA2]$

(12) $\frac{d[\mathrm{SIRT1}]}{dt} = r_{\mathrm{SIRT1}}[sirt1] - \mu_{\mathrm{SIRT1}}[\mathrm{SIRT1}]$

(13) $\frac{d[yy1]}{dt} = s_{yy1} - \delta_{yy1}[yy1] - k_{on3}[yy1] * [miRNA2] + k_{off3}[yy1 - miRNA2]$

(14) $\frac{d[\mathrm{YY1}]}{dt} = r_{\mathrm{YY1}}[yy1] - \mu_{\mathrm{YY1}}[\mathrm{YY1}]$

(15) $\frac{d[miRNA3]}{dt} = s_{miRNA3} + \varepsilon_{miRNA3}\frac{[\mathrm{P53^*}]^4}{[\mathrm{P53^*}]^4+K_3{}^4} - \delta_{miRNA3}[miRNA3] - k_{on4}[cdc42] * [miRNA3] + k_{off4}[cdc42 -$

$miRNA3] - k_{on5}[wip1] * [miRNA3] + k_{off5}[wip1 - miRNA3]$

(16) $\frac{d[cdc42-miRNA3]}{dt} = k_{on4}[cdc42] * [miRNA3] - k_{off4}[cdc42 - miRNA3] - \delta_{c-mi3}[cdc42 - miRNA3]$

(17) $\frac{d[wip1-miRNA3]}{dt} = k_{on5}[wip1] * [miRNA3] - k_{off5}[wip1 - miRNA3] - \delta_{w-mi3}[wip1 - miRNA3]$

(18) $\frac{d[cdc42]}{dt} = s_{cdc42} - \delta_{cdc42}[cdc42] - k_{on4}[cdc42] * [miRNA3] + k_{off4}[cdc42 - miRNA3]$

(19) $\frac{d[\mathrm{CDC42}]}{dt} = r_{\mathrm{CDC42}}[cdc42] - \mu_{\mathrm{CDC42}}[\mathrm{CDC42}]$

(20) $\frac{d[wip1]}{dt} = s_{wip1} + \varepsilon_{wip1}\frac{[\mathrm{P53^*}]^4}{[\mathrm{P53^*}]^4+K_W{}^4} - k_{on5}[wip1] * [miRNA3] + k_{off5}[wip1 - miRNA3]$

(21) $\frac{d[\mathrm{WIP1}]}{dt} = r_{\mathrm{WIP1}}[wip1] - \mu_{\mathrm{WIP1}}[\mathrm{WIP1}]$

(22) $\frac{d[\mathrm{ATM^*}]}{dt} = \beta_{\mathrm{s}}\theta(t) - v_{\mathrm{ATM}}[\mathrm{WIP1}]\frac{[ATM^*]}{[ATM^*]+K_a^*} - \mu_{\mathrm{ATM^*}}[\mathrm{ATM^*}]$

The representation of the 22 biomolecular species used in the ODEs is listed in **Table S2**. In sum, the italic lower-case words represent mRNAs and microRNAs, while the regular upper-case words represent protein species. Next we discuss the key regulations and parameters of the model:

(A) The p53-dependent transcription rate of *mdm2* mRNA and *wip1* mRNA as well as microRNA-192, -34a, and -29a is represented by an *n*th order Hill function of $p53^*$. The Hill coefficient *n* is chosen to be 4 to account for the cooperativity of the tetrameric form of $p53^*$ as a transcription factor [5]. For the microRNA induction, we assume that the p53-dependent

microRNA synthesis rate is 5 times that of the basal synthesis rate, based on previous experimental observations that the p53-induced level of miRNAs is about 5-8 fold higher than their basal induction level [6, 7].

(B) The inhibition of the mRNAs, *mdm2*, *yy1*, *sirt1*, *wip1* and *cdc42*, by a microRNA follows the reaction scheme proposed by Mukherji *et al.* that was experimentally validated [8]. Specifically, the microRNA binds with its target mRNA molecule with high affinity, forming a microRNA-mRNA complex, and subsequently dispose the complex into a degradation machinery.

(C) The mRNAs of YY1, SIRT1 and CDC42, which are intermediate proteins in the microRNA-mediated positive feedback loops but not transactivated by p53, are induced at their respective basal transcription rate.

(D) The post-translational regulations of the P53 and MDM2 proteins, promoted by YY1, SIRT1, Wip1 and CDC42 proteins, are represented by Michaelis-Menten mechanisms or Hill functions. Specifically, SIRT1, Wip1 and CDC42 promote the deactivation of p53, and are thus modeled by Michaelis-Menten mechanisms, where SIRT1, Wip1 and CDC42 serve as the catalysts. The effect of Wip1 repressing the degradation of MDM2 is formulated as an inhibitory Hill function. The effect of YY1 enhancing the MDM2-dependent degradation of p53 is formulated as an activating Hill function. Note that the activating/deactivating regulations of p53 protein through the posttranslational modifications are complicated, including phosphorylation/dephosphorylation etc. Previous studies have shown that p53 can be phosphorylated by ATM directly or indirectly at Ser15, Ser20 and Ser46 in response to ionizing radiation [9, 10]. In our model, we lump these phosphorylation steps into one synergetic activation reaction, which is assumed to be promoted by active ATM. On the other hand, p53 is dephosphorylated by Wip1 at Ser15 directly and at Ser46 indirectly [11, 12]. In addition, SIRT1 and CDC42 also reduce the activity of p53. Analogously, we use a synergetic deactivation reaction in the model, inhibited by Wip1, SIRT1 and CDC42, to account for the multiple deactivating processes.

(E) The formulations of other molecular regulations for the mRNAs and proteins of p53 and MDM2, including the transcription of the *p53* mRNA, the phosphorylation /dephosphorylation of p53 protein, and the degradation of p53 and MDM2 proteins are formatted in the same way as our previous model of p53-MDM2 autoregulatory circuit [1, 13]. The detailed discussion of their biochemical meaning can be found in the supplementary information of [1].

(F) The model parameters for the p53-MDM2 autoregulatory circuit are adapted from [1]. Specifically, the transcription and translation rates are relatively smaller than the post-translational rates. The parameters for the auxiliary regulatory circuits consisting of the three microRNAs and the intermediating proteins, including the transcription rates and translation rates, follow the ranges used for the p53-MDM2 core circuit. In addition, we assume that the degradation rate constants for the mRNAs in the auxiliary circuit are the same and assigned with the value of 0.062 min^{-1}, corresponding to ~11 min half-life, which is the median half-

life of an mRNA measured in recent experiments [14]. The kinetics of microRNA binding to mRNA have been suggested to display rates varying in several orders of magnitude [15, 16]. We assume that, plausibly, the binding of an microRNA to its specific target mRNA is strong and thus the association rate is high, which is indeed an important underlying mechanism inducing the sensitive regulation of mRNA by microRNA supported by experiments [8]. Note that the association and dissociation rate constants of similar scale have been used in previous modeling work [17, 18]. The parameter values of the deterministic single-cell model are listed in **Table S3**.

(G) The formulation of the stimulation of active ATM by DNA damage signal is represented by $\beta_s*\theta(t)$ as proposed in [4], where $\theta(t)$ is the Heaviside step function starting from t=0. The active ATM is inhibited by Wip1, a phosphotase of ATM, thus forming a second negative feedback through Wip1-inducing p53. This inhibition process is represented by a Michaelis-Menten mechanism, as used in a previous model [19].

(H) The microRNA inhibitor used in the experiment has complementary sequence of the target microRNA. Therefore in the model, we assume that each inhibitor binds to its microRNA target and the inhibitor-microRNA complex is subsequently degraded to achieve the repression function. That is, for each inhibitor species there are five accompanying parameters; namely, the synthesis rate, association rate, dissociation rate, degradation rate and the degradation rate for the complex. Since our experiments indicate that the level of miRNAs decreased by ~6 fold when inhibitors were applied, we tuned the binding affinity parameters such that the level of free miRNAs with the inhibitors included in the model declined to be ~6 fold less than that in the wild-type condition. These 15 parameters of the three microRNA inhibitors are listed at the end of **Table S3**.

References:

1. Ma, L., et al., *A plausible model for the digital response of p53 to DNA damage.* Proc Natl Acad Sci U S A, 2005. **102**(40): p. 14266-71.
2. Kim, J.K. and T.L. Jackson, *Mechanisms that enhance sustainability of p53 pulses.* PLoS One, 2013. **8**(6): p. e65242.
3. Batchelor, E., A. Loewer, and G. Lahav, *The ups and downs of p53: understanding protein dynamics in single cells.* Nat Rev Cancer, 2009. **9**(5): p. 371-7.
4. Batchelor, E., et al., *Recurrent initiation: a mechanism for triggering p53 pulses in response to DNA damage.* Mol Cell, 2008. **30**(3): p. 277-89.
5. Jeffrey, P.D., S. Gorina, and N.P. Pavletich, *Crystal structure of the tetramerization domain of the p53 tumor suppressor at 1.7 angstroms.* Science, 1995. **267**(5203): p. 1498-502.
6. Raver-Shapira, N., et al., *Transcriptional activation of miR-34a contributes to p53-mediated apoptosis.* Mol Cell, 2007. **26**(5): p. 731-43.
7. Pichiorri, F., et al., *Downregulation of p53-inducible microRNAs 192, 194, and 215 impairs the p53/MDM2 autoregulatory loop in multiple myeloma development.* Cancer Cell, 2010. **18**(4): p. 367-81.

8. Mukherji, S., et al., *MicroRNAs can generate thresholds in target gene expression.* Nat Genet, 2011. **43**(9): p. 854-9.
9. Kodama, M., et al., *Requirement of ATM for rapid p53 phosphorylation at Ser46 without Ser/Thr-Gln sequences.* Mol Cell Biol, 2010. **30**(7): p. 1620-33.
10. Saito, S., et al., *ATM mediates phosphorylation at multiple p53 sites, including Ser(46), in response to ionizing radiation.* J Biol Chem, 2002. **277**(15): p. 12491-4.
11. Crescenzi, E., et al., *Down-regulation of wild-type p53-induced phosphatase 1 (Wip1) plays a critical role in regulating several p53-dependent functions in premature senescent tumor cells.* J Biol Chem, 2013. **288**(23): p. 16212-24.
12. Lu, X., et al., *The Wip1 Phosphatase acts as a gatekeeper in the p53-Mdm2 autoregulatory loop.* Cancer Cell, 2007. **12**(4): p. 342-54.
13. Wagner, J., et al., *p53-Mdm2 loop controlled by a balance of its feedback strength and effective dampening using ATM and delayed feedback.* Syst Biol (Stevenage), 2005. **152**(3): p. 109-18.
14. Miller, C., et al., *Dynamic transcriptome analysis measures rates of mRNA synthesis and decay in yeast.* Mol Syst Biol, 2011. **7**: p. 458.
15. Morozova, N., et al., *Kinetic signatures of microRNA modes of action.* Rna, 2012. **18**(9): p. 1635-55.
16. Zinovyev, A., et al., *Mathematical Modeling of microRNA-Mediated Mechanisms of Translation Repression*, in *MicroRNA Cancer Regulation: Advanced Concepts, Bioinformatics and Systems Biology Tools,*, U. Schmitz, O. Wolkenhauer, and J. Vera, Editors. 2013, Springer. p. 189-224.
17. Hoffmann, A., et al., *The IkappaB-NF-kappaB signaling module: temporal control and selective gene activation.* Science, 2002. **298**(5596): p. 1241-5.
18. Malek, S., T. Huxford, and G. Ghosh, *Ikappa Balpha functions through direct contacts with the nuclear localization signals and the DNA binding sequences of NF-kappaB.* J Biol Chem, 1998. **273**(39): p. 25427-35.
19. Zhang, X.P., F. Liu, and W. Wang, *Two-phase dynamics of p53 in the DNA damage response.* Proc Natl Acad Sci U S A, 2011. **108**(22): p. 8990-5.
