# Supplementary material for: MiR-192-Mediated Positive Feedback Loop Controls the Robustness of Stress-Induced p53 Oscillations in Breast Cancer Cells
Source: PLoS Comput Biol. 2015 Dec 7;11(12):e1004653. doi: 10.1371/journal.pcbi.1004653 (PMC4671655; doi:10.1371/journal.pcbi.1004653)
Supplement: S2 Text — (PDF) [file pcbi.1004653.s002.pdf]

S2 Text. Robustness analysis of the single-cell model of microRNA-p53-MDM2 network

To further quantify the impact of different types of microRNAs on regulating the p53 network, we measure the system's robustness performance, which is the capability to maintain the oscillatory behavior of p53 in response to DNA damage. In particular, we use the parametric oscillation range to quantify the degree of system robustness. Since under a microRNA perturbation, the parametric oscillation range may shift drastically such that the nominal value of the wild-type model is no longer inside it, the smallest distance of the individual oscillation boundary point relative to the nominal parameter (assuming always positive) as used previously [1, 2] is not a meaningful metric any more for the robustness study here. We use the oscillation range normalized by the nominal parameter value as the robustness index (RI) of the model:

$$RI \triangleq \frac{k_i^{UB} - k_i^{LB}}{k_i^*},$$

where k_i^* is the wild-type nominal parameter value, k_i^{LB} and k_i^{UB} are the lower and upper bound of the parametric oscillation region, respectively, for the selected parameter under different cellular scenarios. Intuitively, a large robustness index indicates higher robustness and consequently less sensitivity of the system behavior to the perturbation of extrinsic noise and vice versa. The robustness indices thus calculated for the wild-type case and the microRNA-repressed cases with respect to the 14 parameters are listed in **Table S7**, allowing for direct comparison of the impact of different microRNA inhibition. Note that for several cases our computation indicates that the oscillation upper bound $k_i^{LB}$is unlimited, and thus the value of RI is infinity. For these scenarios, if the lower bound for oscillation k_i^{LB} exists, we should then compare the values of k_i^{LB} to determine the relative robustness, where a larger k_i^{LB}indicates smaller oscillation range and hence less robustness. The results in **Table S7** show that overall the wild-type p53-MDM2-microRNA system has the highest RI, while the system under the repression of miR-192 has the lowest RI.

As a summary, the robustness index agrees with the qualitative read-out of the bifurcation diagrams that the oscillatory phenotype when miR-192 is repressed is the least robust among the three cases. Specifically, the inhibition of miR-192 drastically undermines the robust sustainability of p53 oscillation and yields a considerably smaller oscillatory region. The inhibition of miR-34a and miR-29a only leads to mildly reduced or similar oscillatory sustainability compared to that of the wild-type case. Thus we infer that the inhibition of miR-192 will lead to the highest probability of non-oscillating cells in a population due to extrinsic noise.

References

1. Ma, L. and P.A. Iglesias, *Quantifying robustness of biochemical network models.* BMC Bioinformatics, 2002. **3**: p. 38.
2. Ma, L. and R. Ranganathan, *Systems-level characterization of the kernel mechanism of the cyanobacterial circadian oscillator.* Biosystems, 2014. **117**: p. 30-9.
