# Supplementary material for: MiR-192-Mediated Positive Feedback Loop Controls the Robustness of Stress-Induced p53 Oscillations in Breast Cancer Cells
Source: PLoS Comput Biol. 2015 Dec 7;11(12):e1004653. doi: 10.1371/journal.pcbi.1004653 (PMC4671655; doi:10.1371/journal.pcbi.1004653)
Supplement: S1 Table — (PDF) [file pcbi.1004653.s003.pdf]

S1 Table. List of the 22 molecular species used in the deterministic model.

**Species Name**	**Species Description**
$p53$	*p53* mRNA
$mdm2$	*mdm2* mRNA
P53	Inactive P53 protein
$P53^*$	Active P53 protein
MDM2	MDM2 protein
$miRNA1$	microRNA-192
$mdm2-miRNA1$	Complex of *mdm*2 mRNA and *microRNA*-192
$miRNA2$	microRNA-34a
$sirt1-miRNA2$	Complex of *sirt*1 mRNA and *microRNA*-34a
$yy1-miRNA2$	Complex of *yy*1 mRNA and *microRNA*-34a
$miRNA3$	microRNA-29a
$cdc42-miRNA3$	Complex of *cdc4*2 mRNA and *microRNA*-29a
$wip1-miRNA3$	Complex of *wip*1 mRNA and *microRNA*-29a
$sirt1$	*sirt*1 mRNA
SIRT1	SIRT1 protein
$yy1$	*yy*1 mRNA
YY1	YY1 protein
$cdc42$	*cdc42* mRNA
CDC42	CDC42 protein
$wip1$	*wip1* mRNA
WIP1	Wip1 protein
ATM^*	Active ATM monomer
