# Supplementary material for: MiR-192-Mediated Positive Feedback Loop Controls the Robustness of Stress-Induced p53 Oscillations in Breast Cancer Cells
Source: PLoS Comput Biol. 2015 Dec 7;11(12):e1004653. doi: 10.1371/journal.pcbi.1004653 (PMC4671655; doi:10.1371/journal.pcbi.1004653)
Supplement: S2 Table — (PDF) [file pcbi.1004653.s004.pdf]

S2 Table. List of the 90 parameters used in the deterministic model.

Parameter	Description	Units	Constant
s_{p53}	Basal induction rate of *p53* mRNA	μM·min^{-1}	2.4x10^{-5}
s_{mdm2}	Basal induction rate of *mdm2* mRNA	μM·min^{-1}	2x10^{-5}
ε_{mdm2}	P53*-dependent *mdm2* transcription rate	μM·min^{-1}	0.05
r_{P53}	Translation rate of p53	min^{-1}	1.1
r_{MDM2}	Translation rate of MDM2	min^{-1}	0.42
δ_{p53}	Degradation rate of *p53* mRNA	min^{-1}	0.002
δ_{mdm2}	Degradation rate of *Mdm2* mRNA	min^{-1}	0.0037
μ_{P53}	Basal degradation rate of P53	min^{-1}	0.02
μ_{MDM2}	Basal degradation rate of MDM2	min^{-1}	0.007
ν_{P53}	MDM2-dependent degradation rate of P53	min^{-1}	0.085
k_v	Ratio of MDM2-dependent degradation rate of P53over that of P53*	unitless	5
ν_{MDM2}	ATM*-dependent degradation rate of Mdm2	min^{-1}	0.05
k_{fp}	Phosphorylation (activation) rate of P53	min^{-1}	15
k_{rp}	Dephosphorylation (deactivation) rate of P53*	min^{-1}	1.35
K_m	Michaelis constant of p53-dependent *mdm2* transcription	μM	0.7
K_d	Threshold conc. for Mdm2-dependent P53 degradation	μM	0.0261
K_d^*	Threshold conc. for MDM2-dependent P53* degradation	μM	0.261
K_p	Michaelis constant of ATM*-dependent P53 phosphorylation	μM	0.87
K_a	Threshold conc. for ATM*-dependent MDM2 degradation	μM	1.5
k_{on1}	Association rate between *mdm*2 mRNA and *miRNA*1	μM^{-1}·min^{-1}	25
k_{off1}	Dissociation rate of *mdm*2-*miRNA*1 complex	min^{-1}	0.13
k_w	Factor of Wip1-dependent decreased degradation of MDM2	unitless	1
K_w	Michaelis constant of WIP1 dependent degradation of MDM2	μM	0.08
s_{miRNA1}	Basal induction rate of *miRNA*1	μM·min^{-1}	2.4x10^{-5}
ε_{miRNA1}	P53*-induced transcription rate of *miRNA*1	μM·min^{-1}	0.000125

K_1	Michaelis constant of $P53^*$-dependent *miRNA*1 transcription	μM	0.001
δ_{miRNA1}	Degradation rate of *miRNA*1	min^{-1}	0.0078
δ_{M-mi1}	Degradation rate of *mdm*2-*miRNA*1 complex	min^{-1}	0.062
s_{miRNA2}	Basal induction rate of *miRNA*2	$\mu M \cdot min^{-1}$	$2.4 x 10^{-5}$
ε_{miRNA2}	$P53^*$-induced transcription rate of *miRNA*2	$\mu M \cdot min^{-1}$	0.000125
K_2	Michaelis constant of $P53^*$-dependent *miRNA*2 transcription	μM	0.001
δ_{miRNA2}	Degradation rate of *miRNA*2	min^{-1}	0.0078
s_{sirt1}	Basal induction rate of *sirt*1 mRNA	$\mu M \cdot min^{-1}$	$1.4 x 10^{-5}$
δ_{sirt1}	Degradation rate of *sirt*1 mRNA	min^{-1}	0.062
k_{on2}	Association rate between *sirt*1mRNA and *miRNA*2	$\mu M^{-1} \cdot min^{-1}$	25
k_{off2}	Dissociation rate of *sirt*1-*miRNA*2 complex	min^{-1}	0.13
δ_{s-mi2}	Degradation rate of *sirt*1- *miRNA*2 complex	min^{-1}	0.062
r_{SIRT1}	Translation rate of SIRT1 protein	min^{-1}	0.42
μ_{SIRT1}	Degradation rate of SIRT1 protein	min^{-1}	0.03
s_{yy1}	Basal induction rate of *yy*1mRNA	$\mu M \cdot min^{-1}$	$1.4 x 10^{-5}$
δ_{yy1}	Degradation rate of *yy*1mRNA	min^{-1}	0.062
k_{on3}	Association rate between *yy*1mRNA and *mRNA*2	$\mu M^{-1} \cdot min^{-1}$	25
k_{off3}	Dissociation rate of *yy*1-*miRNA*2 complex	min^{-1}	0.13
δ_{y-mi2}	Degradation rate of *yy*1-*miRNA*2 complex	min^{-1}	0.062
r_{YY1}	Translation rate of YY1 protein	min^{-1}	0.75
μ_{YY1}	Degradation rate of YY1 protein	min^{-1}	0.03
s_{miRNA3}	Basal induction rate of *miRNA*3	$\mu M \cdot min^{-1}$	$2.4 x 10^{-5}$
ε_{miRNA3}	$P53^*$-induced transcription rate of *miRNA*3	$\mu M \cdot min^{-1}$	0.000125
K_3	Michaelis constant of $P53^*$-dependent *miRNA*3 transcription	μM	0.001
δ_{miRNA3}	Degradation rate of *miRNA*3	min^{-1}	0.0078
δ_{c-mi3}	Degradation rate of *cdc*42-*miRNA*3 complex	min^{-1}	0.062
s_{cdc42}	Basal induction rate of *cdc*42 mRNA	$\mu M \cdot min^{-1}$	$1.4 x 10^{-5}$
δ_{cdc42}	Degradation rate of *cdc*42 mRNA	min^{-1}	0.062
k_{on4}	Association rate between *cdc*42mRNA and *miRNA*3	$\mu M^{-1} \cdot min^{-1}$	25

k_{off4}	Dissociation rate of *cdc*42-*miRNA*3 complex	min^{-1}	0.13
δ_{c-mi3}	Degradation rate of *cdc*42-*miRNA*3 complex	min^{-1}	0.062
r_{CDC42}	Translation rate of CDC42 protein	min^{-1}	0.42
μ_{CDC42}	Degradation rate of CDC42 protein	min^{-1}	0.03
s_{wip1}	Basal induction rate of *wip*1 mRNA	$\mu M \cdot min^{-1}$	$2.4x10^{-5}$
δ_{wip1}	Degradation rate of *wip*1 mRNA	min^{-1}	0.062
k_{on5}	Association rate between *wip*1mRNA and *miRNA*3	$\mu M^{-1} \cdot min^{-1}$	25
k_{off5}	Dissociation rate of *wip*1-*miRNA*3 complex	min^{-1}	0.13
δ_{w-mi3}	Degradation rate of *wip*1-*miRNA*3 complex	min^{-1}	0.062
r_{WIP1}	Translation rate of WIP1 protein	min^{-1}	0.42
μ_{WIP1}	Degradation rate of WIP1 protein	min^{-1}	0.03
k_{da1}	Deactivation rate of P53* by SIRT1	min^{-1}	3.3
k_{da2}	Deactivation rate of P53* by CDC42	min^{-1}	3.3
k_{da3}	Deactivation rate of P53* by WIP1	min^{-1}	3.3
K_{da1}	Michaelis constant of SIRT1-dependent P53 deactivation	μM	0.0087
K_{da2}	Michaelis constant of CDC42-dependent P53 deactivation	μM	0.0087
K_{da3}	Michaelis constant of WIP1-dependent P53 deactivation	μM	0.0087
k_{yy1}	Enhancement factor of MDM2-dependent degradation of P53 /$P53^*$ by YY1	unitless	3.5
K_{yy1}	Hill constant of YY1-promoting MDM2-dependent degradation of P53 /$P53^*$	μM	0.01
ε_{wip1}	$P53^*$-dependent *wip1* transcription rate	$\mu M \cdot min^{-1}$	0.000125
K_w	Michaelis constant of p53-dependent *wip1* transcription	μM	0.1
v_{ATM}	WIP1-dependent degradation rate of ATM^*	min^{-1}	30
K_a	Threshold conc. for WIP1-dependent ATM^* degradation	μM	0.1
β_s	DNA damage signal production rate	$\mu M \cdot min^{-1}$	1
s_{inh1}	Synthesis rate of inhibitor1	$\mu M \cdot min^{-1}$	$1.4x10^{-4}$
$k_{on-inh1}$	Association rate between inhibitor1 and *miRNA*1	$\mu M^{-1} \cdot min^{-1}$	8.5
$k_{off-inh1}$	Dissociation rate of inhibitor1-*miRNA*1 complex	min^{-1}	0.028
δ_{inh1}	Degradation rate of inhibitor1	min^{-1}	0.008

$\delta_{inh1-mi1}$	Degradation rate of inhibitor1-*miRNA*1 complex	min^{-1}	0.0625
s_{inh2}	Synthesis rate of inhibitor2	$\mu M \cdot min^{-1}$	$1.4 x 10^{-4}$
$k_{on-inh2}$	Association rate between inhibitor2 and *miRNA*2	$\mu M^{-1} \cdot min^{-1}$	25
$k_{off-inh2}$	Dissociation rate of inhibitor2-*miRNA*2 complex	min^{-1}	0.028
δ_{inh2}	Degradation rate of inhibitor2	min^{-1}	0.008
$\delta_{inh2-mi2}$	Degradation rate of inhibitor2-*miRNA*2 complex	min^{-1}	0.0625
s_{inh3}	Synthesis rate of inhibitor3	$\mu M \cdot min^{-1}$	$1.38 x 10^{-4}$
$k_{on-inh3}$	Association rate between inhibitor3 and *miRNA*3	$\mu M^{-1} \cdot min^{-1}$	25
$k_{off-inh3}$	Dissociation rate of inhibitor3-*miRNA*3 complex	min^{-1}	0.028
δ_{inh3}	Degradation rate of inhibitor3	min^{-1}	0.008
$\delta_{inh3-mi3}$	Degradation rate of inhibitor3-*miRNA*3 complex	min^{-1}	0.0625
