# Supplementary material for: MiR-192-Mediated Positive Feedback Loop Controls the Robustness of Stress-Induced p53 Oscillations in Breast Cancer Cells
Source: PLoS Comput Biol. 2015 Dec 7;11(12):e1004653. doi: 10.1371/journal.pcbi.1004653 (PMC4671655; doi:10.1371/journal.pcbi.1004653)
Supplement: S3 Table — (PDF) [file pcbi.1004653.s005.pdf]

S3 Table. List of the fourteen parameters along the microRNA-based positive feedback loops that are subject to bifurcation analysis in the main Text.

Parameter	Description
ε_{miRNA1}	P53*-induced transcription rate of *microRNA*1 (miR-192)
ε_{miRNA2}	P53*-induced transcription rate of *microRNA*2 (miR-34a)
ε_{miRNA3}	P53*-induced transcription rate of *microRNA*3 (miR-29a)
k_{da1}	Deactivation rate of P53* by SIRT1
k_{da2}	Deactivation rate of P53* by CDC42
k_{da3}	Deactivation rate of P53* by Wip1
k_{on1}	Association rate between *mdm*2 mRNA and *microRNA*1
k_{on2}	Association rate between *sirt*1mRNA and *microRNA*2
k_{on3}	Association rate between *yy*1mRNA and *microRNA*2
k_{on4}	Association rate between *cdc*42mRNA and *microRNA*3
k_{on5}	Association rate between *wip*1mRNA and *microRNA*3
k_w	Factor of Wip1-dependent decreased degradation of MDM2
k_{yy1}	Enhancement factor of MDM2-dependent degradation of P53 /P53* by YY1
v_{P53}	MDM2-dependent degradation rate of P53
