# Supplementary material for: MiR-192-Mediated Positive Feedback Loop Controls the Robustness of Stress-Induced p53 Oscillations in Breast Cancer Cells
Source: PLoS Comput Biol. 2015 Dec 7;11(12):e1004653. doi: 10.1371/journal.pcbi.1004653 (PMC4671655; doi:10.1371/journal.pcbi.1004653)
Supplement: S4 Table — The abbreviation “wrt” denotes “with respect to”. (PDF) [file pcbi.1004653.s006.pdf]

S4 Table. The Robustness Index (RI) of the microRNA-p53-MDM2 system with respect to the fourteen selected parameters under the wild-type and microRNA-repressed conditions. The abbreviation "*wrt*" denotes "with respect to".

System Condition	RI *wrt* $\varepsilon_{\mathrm{miRNA1}}$	RI *wrt* $\varepsilon_{\mathrm{miRNA2}}$	RI *wrt* $\varepsilon_{\mathrm{miRNA3}}$	RI *wrt* k_{da1}	RI *wrt* k_{da2}	RI *wrt* k_{da3}	RI *wrt* k_{on1}	RI *wrt* k_{on2}	RI *wrt* k_{on3}	RI *wrt* k_{on4}	RI *wrt* k_{on5}
Wild Type	11.2	9	∞	∞	∞	∞	∞	∞	∞	∞	2.4
miR-192 repressed	13.2	0	0	∞	∞	18	∞	0	0.8	0	0.4
miR-34a repressed	10.8	14.4	∞	∞	∞	∞	∞	∞	∞	∞	∞
miR-29a repressed	9.6	10	∞	∞	∞	18.2	∞	∞	∞	∞	2.56

System Condition	RI *wrt* k_{w}	RI *wrt* k_{yy1}	RI *wrt* v_{p53}
Wild Type	1.9	7.32	∞
miR-192 repressed	0.5	0	0
miR-34a repressed	2.4	3.3	∞
miR-29a repressed	2.6	7	∞
