# Supplementary material for: MiR-192-Mediated Positive Feedback Loop Controls the Robustness of Stress-Induced p53 Oscillations in Breast Cancer Cells
Source: PLoS Comput Biol. 2015 Dec 7;11(12):e1004653. doi: 10.1371/journal.pcbi.1004653 (PMC4671655; doi:10.1371/journal.pcbi.1004653)
Supplement: S5 Table — (PDF) [file pcbi.1004653.s007.pdf]

S5 Table. Percentage of cells that oscillate following treatment with microRNA inhibitor in duplicate experiments.

		Wildtype	FF4	miR-192 Inhibited	miR-34a Inhibited	miR-29a Inhibited
Oscillating Cells (%)	Experiment 1	68	72	54	71	65
	Experiment 2	83	89	65	81	79
