# Supplementary material for: MiR-192-Mediated Positive Feedback Loop Controls the Robustness of Stress-Induced p53 Oscillations in Breast Cancer Cells
Source: PLoS Comput Biol. 2015 Dec 7;11(12):e1004653. doi: 10.1371/journal.pcbi.1004653 (PMC4671655; doi:10.1371/journal.pcbi.1004653)
Supplement: S1 Fig — A paired dots represent the bounds of p53 oscillation amplitude and the solid line represents stationary steady state. The red vertical line indicates the nominal parameter value. Again, the inhibition of miR-192 leads to shrunk oscillating region or completely non-oscillating region over varying parameter ranges, while the inhibition of miR-34a and miR-29a mildly affects the systems behavior. (PDF) [file pcbi.1004653.s009.pdf]

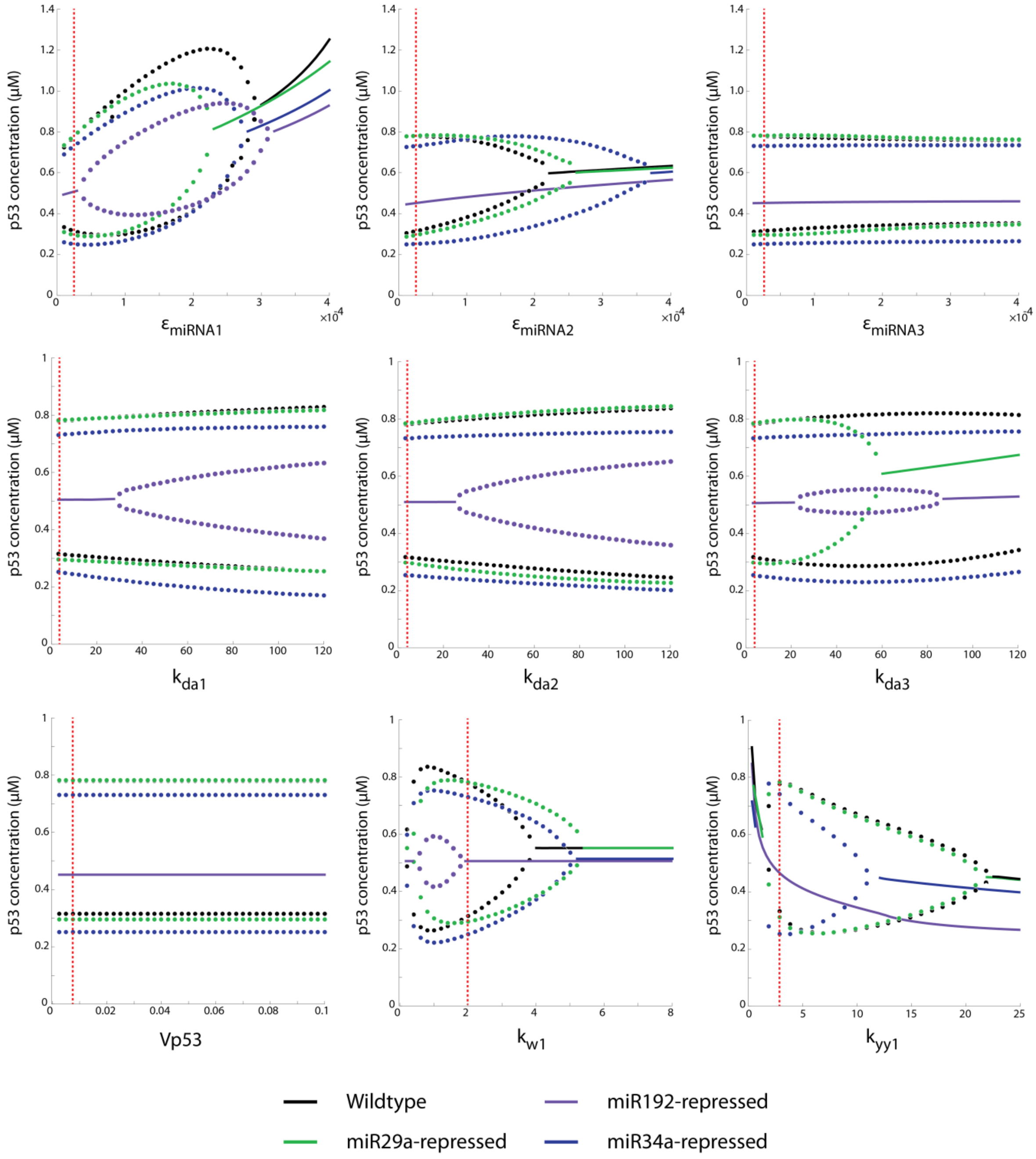

S1 Fig. Bifurcation diagrams of the DNA double-strand-break triggered steady-state p53 response versus 9 parameters (ε_{miRNA1}, ε_{miRNA2}, ε_{miRNA3}, k_{da1}, k_{da2}, k_{da3}, k_{w1}, v_{p53} and k_{yy1}) embedded in the positive feedback loops, under wild-type (black), miR-192-repressed (purple), miR-34a-repressed (blue), and miR-29a-repressed (green) conditions. A paired dots represent the bounds of p53 oscillation amplitude and the solid line represents stationary steady state. The red vertical line indicates the nominal parameter value. Again, the inhibition of miR-192 leads to shrunk oscillating region or completely non-oscillating region over varying parameter ranges, while the inhibition of miR-34a and miR-29a mildly affects the systems behavior.
