# Supplementary material for: MiR-192-Mediated Positive Feedback Loop Controls the Robustness of Stress-Induced p53 Oscillations in Breast Cancer Cells
Source: PLoS Comput Biol. 2015 Dec 7;11(12):e1004653. doi: 10.1371/journal.pcbi.1004653 (PMC4671655; doi:10.1371/journal.pcbi.1004653)
Supplement: S2 Fig — Through this step, the raw signal of p53 trajectory is decomposed into a smooth denoised signal and high-frequency residuals. (PDF) [file pcbi.1004653.s010.pdf]

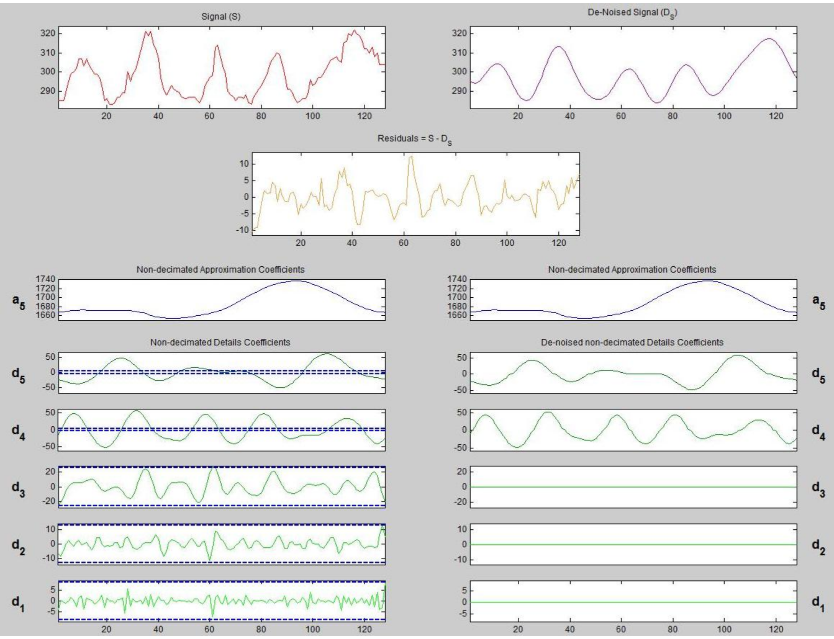

S2 Fig: MATLAB GUI (graphical user interface) of the Stationary Wavelet Transform 1-D tool. Through this step, the raw signal of p53 trajectory is decomposed into a smooth denoised signal and high-frequency residuals.
