# Supplementary material for: MiR-192-Mediated Positive Feedback Loop Controls the Robustness of Stress-Induced p53 Oscillations in Breast Cancer Cells
Source: PLoS Comput Biol. 2015 Dec 7;11(12):e1004653. doi: 10.1371/journal.pcbi.1004653 (PMC4671655; doi:10.1371/journal.pcbi.1004653)
Supplement: S3 Fig — After denoising, peaks and instantaneous slope of each single-cell tracking data obtained before determining whether the observed p53 fluorescence expression was considered oscillatory. Each trajectory is tested for following conditions to determine whether it is oscillatory: 1) less than 3 peaks were detected during the time-lapse, 2) there were 2 consecutive peaks that occur within 5 time units (100 minutes), 3) there were 2 consecutive peaks that occur more than 70 time units (1400 minutes) apart, and 4) the slope of the trajectory was negative (or positive) more than 75% of the time. Trajectories of cells that exhibited criteria 1, 2 and 3 are considered non-oscillating and are shaded in grey. Cells that fall into category 4 are shaded in pink. Number below each graph indicates the ratio of time in which the slope of trajectory is negative. (PDF) [file pcbi.1004653.s011.pdf]

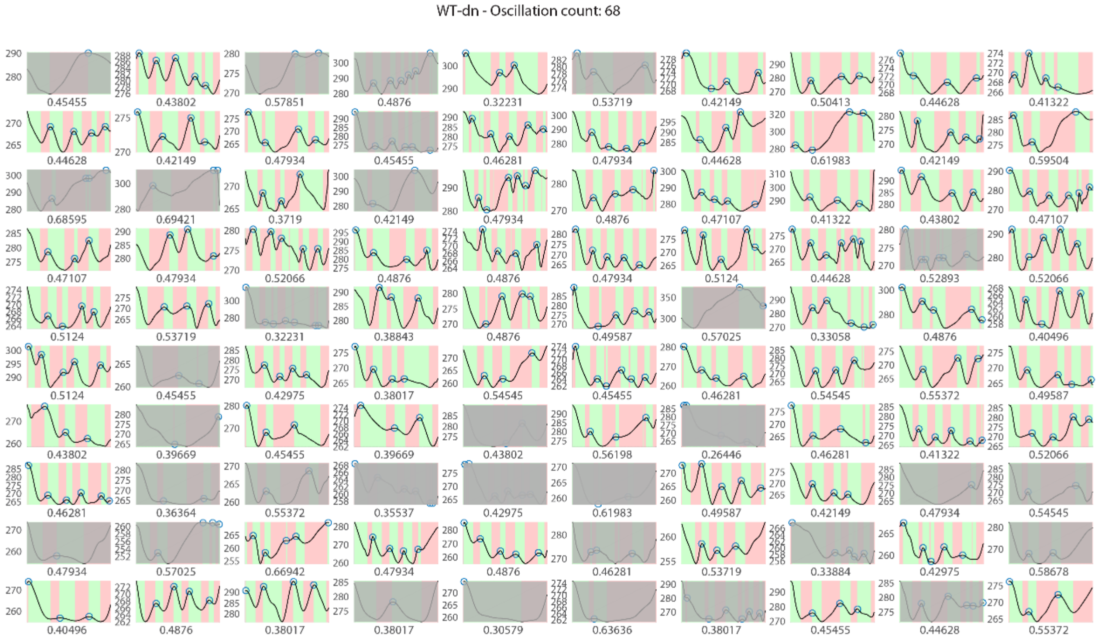

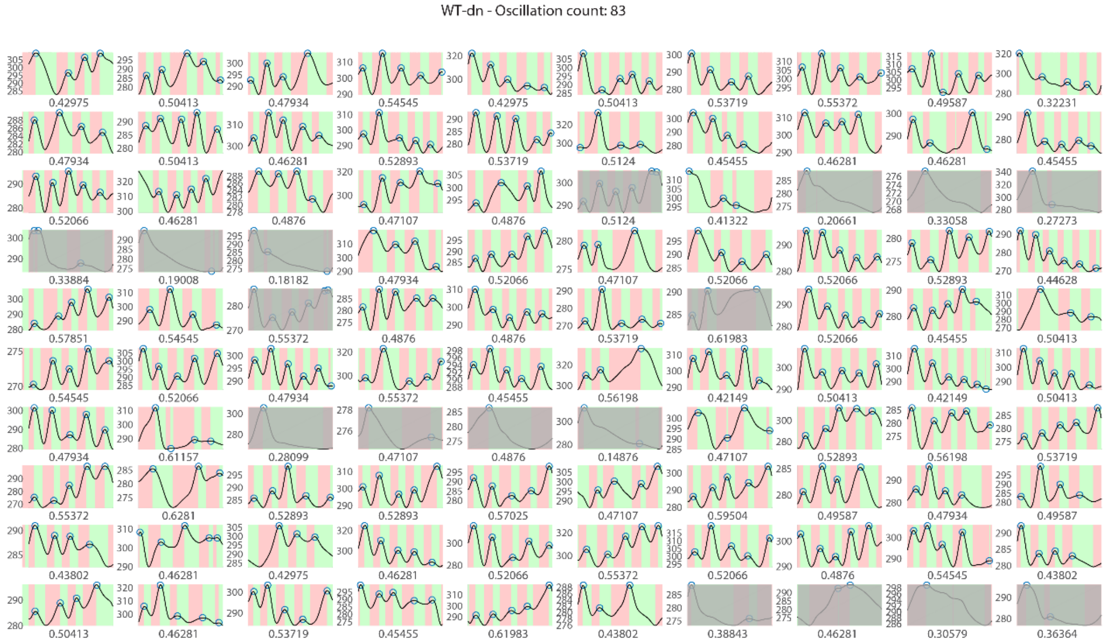

S3 Fig. Detection of oscillation in wild type p53 fluorescence trajectory in duplicate experiments. After denoising, peaks and instantaneous slope of each single-cell tracking data obtained before determining whether the observed p53 fluorescence expression was considered oscillatory. Each trajectory is tested for following conditions to determine whether it is oscillatory: 1) less than 3 peaks were detected during the time-lapse, 2) there were 2 consecutive peaks that occur within 5 time units (100 minutes), 3) there were 2 consecutive peaks that occur more than 70 time units (1400 minutes) apart, and 4) the slope of the trajectory was negative (or positive) more than 75% of the time. Trajectories of cells that exhibited criteria 1, 2 and 3 are considered non-oscillating and are shaded in grey. Cells that fall into category 4 are shaded in pink. Number below each graph indicates the ratio of time in which the slope of trajectory is negative.
