# Supplementary material for: MiR-192-Mediated Positive Feedback Loop Controls the Robustness of Stress-Induced p53 Oscillations in Breast Cancer Cells
Source: PLoS Comput Biol. 2015 Dec 7;11(12):e1004653. doi: 10.1371/journal.pcbi.1004653 (PMC4671655; doi:10.1371/journal.pcbi.1004653)
Supplement: S6 Fig — See S3 Fig for a detailed description. (PDF) [file pcbi.1004653.s014.pdf]

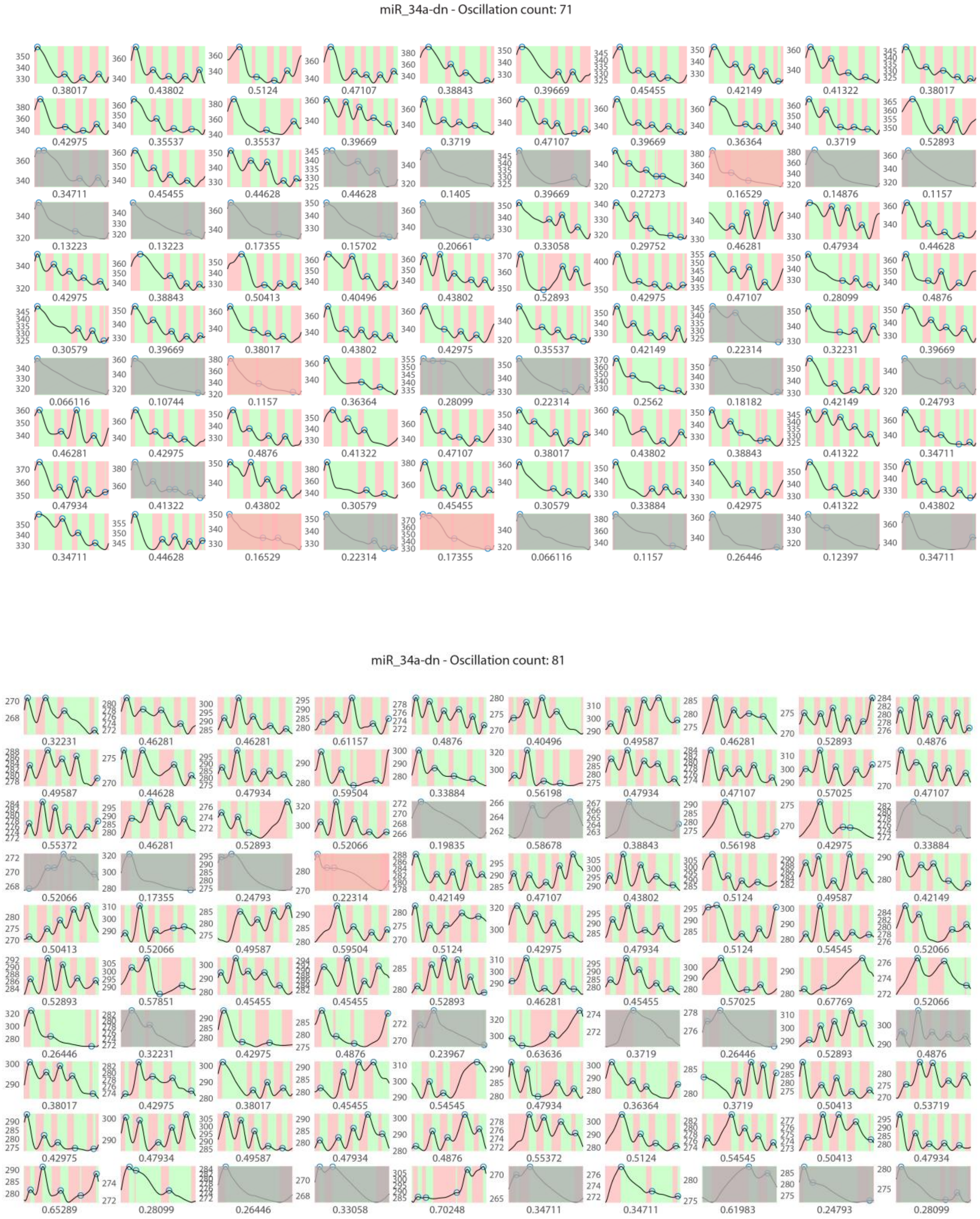

S6 Fig. Detection of oscillation in mir34a-inhibited p53 fluorescence trajectory in duplicate experiments. See **S3 Fig** for a detailed description.
